# Associations between early childhood poverty and cognitive functioning throughout childhood and adolescence: A 14-year prospective longitudinal analysis of the Mauritius Child Health Project

**DOI:** 10.1371/journal.pone.0278618

**Published:** 2023-02-24

**Authors:** Hannah Strauß, Peter Venables, Marcel Zentner

**Affiliations:** 1 Personality, Emotion and Music Laboratory, Department of Psychology, University of Innsbruck, Innsbruck, Austria; 2 Department of Psychology, University of York, York, United Kingdom; University of Padova, ITALY

## Abstract

Associations between childhood poverty and cognitive outcomes have been examined from multiple perspectives. However, most evidence is based on cross-sectional data or longitudinal data covering only segments of the developmental process. Moreover, previous longitudinal research has mostly relied on data from Western nations, limiting insights of poverty dynamics in low- and middle-income countries. Here, we use data from the Mauritius Child Health Project, a large-scale prospective longitudinal study conducted in a then low-income country, to examine long-term associations between poverty in early childhood and cognitive performance across childhood and adolescence. Poverty-related factors were assessed at age 3 years and comprised indicators of psychosocial adversity and malnutrition. Cognitive functioning was assessed at ages 3 and 11 years by using standardized intelligence measures and at age 17 years by means of a computerized test battery. Using multiple hierarchical regression models, we found that chronic malnutrition and parental characteristics showed similar-sized, independent associations with initial cognitive functioning at age 3 as well as at age 11 years. For age 17 years, however, associations with early childhood risk factors vanished and instead, cognitive functioning was predicted by performance on prior cognitive assessments. Sex was also found to be a powerful predictor of cognitive trajectories, with boys improving and girls worsening over time, regardless of the level of their initial exposure to risk. The current findings indicate that, to prevent cognitive impairment, interventions tackling poverty and malnutrition should focus on the infancy period and be designed in a gender-sensitive way.

## Introduction

Research over the past decades has shed important light on the effects of poverty-related adversity on cognitive functioning. It has been shown that differences in IQ scores between children from high- and low-income families already emerge in late infancy and almost triple by adolescence [1]. Overall, children who experienced poverty during their first years of life fall behind those from more advantaged backgrounds by up to 1 *SD* in standardized measures of intelligence or education when they reach adolescence [1, 2]. The lower educational and occupational attainment resulting from these downward trajectories tends to perpetuate an intergenerational poverty cycle [3].

While adverse outcomes of poverty are firmly established, the processes that lead from poverty to its adverse consequences over time are less well understood. For one thing, poverty is often defined at a low level of granularity via basic monetary indicators that do not consider specific poverty-related social and biological (risk) factors, such as malnutrition or psychological stressors, that impact cognition (e.g., [4]). Unsurprisingly, the direct effect of monetary poverty on intellectual development vanishes after poverty-related variables such as home environment, health status of the child, and maternal cognitive abilities are accounted for [5]. For example, in a sample of 1,586 South African children ages 6–8 years, preschool education and nutrition but not SES or maternal education directly predicted children’s cognitive performance [6]. Likewise, analyses of Kenyan toddlers and schoolchildren found that better-nourished children showed higher cognitive performance, independent from the family’s economic resources [7]. Overall, a meta-analysis of studies published in the 1990s based on US children aged 5 to 18 years showed that the size of associations between SES and academic achievement varied depending on the type of SES component [8]. Whereas parental income showed a mean effect size of *r* = .29, similar to the mean effect sizes of parental education and occupation (*r* = .30 and *r =* .28, respectively), the effect size of home resources (e.g., household possessions, cognitive stimulation, parent-child interaction; see [5, 9]) was *r* = .51. These findings indicate that it is crucial to incorporate a variety of co-occurring contextual factors associated with monetary poverty in order to gain insight into its developmental repercussions [8, 10].

However, capturing the multidimensionality of poverty remains a major challenge for researchers and policy makers alike. One way of addressing this issue has been to code individual risk factors as either present or absent and to combine them into cumulative risk indexes that can then be related to developmental outcomes [4]. From the available evidence, it would seem that the more risk factors children experience, the lower their performance on cognitive tests (e.g., [2, 11, 12]). This approach is sensible but also has its limitations. A binary assessment of risk factors as present or absent may miss important gradations within a given risk category, and the assumption that cumulative risk factors act additively may not always be warranted [13].

The understanding and assessment of psychologically consequential agents of poverty aside, another difficulty for research consists of integrating assessments from different developmental stages. Evidence about long-term outcomes associated with early childhood poverty often rests on cross-sectional or retrospective studies and the number of early-onset prospective longitudinal studies extending beyond adolescence remains exceedingly small [1]. Without prospective studies documenting the temporal sequence of cognitive functioning as it relates to poverty, it is difficult to understand how poverty-related adversity affects cognitive outcomes over time.

A final limitation in current research on long-term cognitive consequences of poverty is its reliance on samples from industrialized Western countries. It is often assumed that fundamental principles of optimal development apply to all human beings independent of their cultural and ethnic backgrounds. Yet, samples from low- and middle-income countries show considerable differences from Western populations [14]. First, children from low- and middle-income countries are more likely to be malnourished and to have infectious diseases than are their peers from high-income countries. For example, in 2021 more than four out of five children under 5 years affected by stunted growth—caused by caloric sparsity and infections—lived in low- or lower-middle income countries [15]. Numbers thereby are particularly high for heavily-indebted Sub-Saharan countries (e.g., Chad, Sierra Leone), to which the present sample from Mauritius of the 1970s and 1980s can be roughly compared in terms of GDP per capita. Examining poverty’s effects on cognitive development in a non-Western sample thus provides a valuable addition to previous findings, particularly when it comes to long-term effects of poverty. A second difference between many rich Western and poorer non-Western countries can be found in the latter’s gender-unequal social structures and roles. Although gender inequality is pervasive across the world, it is particularly pronounced across many developing nations [16], where girls are often denied opportunities for education for a variety of reasons, ranging from prejudice based on gender stereotypes at home, at school, and in the community to early and forced marriages. The associations between gender discrimination and cognitive outcomes have been amply documented in cross-sectional research. For example, girls’ lower performance on spatial and mathematical tasks is most pronounced in gender-unequal nations but disappears at the rate of rising gender equality (e.g., [17, 18]). To our knowledge, at present only one prospective longitudinal study has examined sex differences in low- and middle-income countries (i.e., Ethiopia, India, Peru, and Vietnam) [14, 19]. The results showed that although gender differences in cognitive functioning are absent at preschool age, gaps emerge later and widen particularly during adolescence, favoring boys in three of the four countries [19]. We were interested in examining whether these results would replicate in a study conducted in a different low-income nation and at a different time.

To redress some of the aforementioned limitations, we used data from the Mauritius Child Health Project (MCHP). The MCHP is a prospective longitudinal study based on a 1969 birth cohort of 1,795 children. Its primary aim was to identify individuals with high risks of developing mental disorders prior to their onset and to examine the efficacy of early experimental interventions in terms of neurophysiological risk factors. More detailed information on the study design of the MCHP can be found elsewhere [20]. Mauritius, a small island located in the Indian Ocean east of Madagascar, was chosen as the study location because of its small size and low emigration, which facilitated repeated assessment of participants [20]. Gaining independence from Great Britain in 1968, Mauritius faced high unemployment, high population growth, persistent inflation, commodity dependency, increasing external debt, corruption, extreme racial inequality, and low levels of human development throughout the 1970s. It is only in subsequent decades that it has achieved sustained economic progress and reduced inequality, becoming a showcase nation with an average GDP growth of 4.6% [21]. The MCHP thus provides a rare opportunity to examine the associations of poverty and cognitive performance prospectively and over a period that ranges from early childhood to late adolescence in a non-Western sample affected by high levels of poverty at the time of the study. From the aforementioned literature, we assumed that long-term associations between poverty-related risk factors in early childhood and cognitive functioning in adolescence would be driven in particular by parental educational attainment and malnutrition. Also, we assumed to find gender differences emerging in cognitive functioning over time.

## Materials and methods

### Participants

Unlike studies of most other birth cohorts, the MCHP mainly comprises lower and middle-income families who participated in return for free flour and sweets, as well as medical examinations for the children. The birth cohort consisted of a total of 1,795 children born in two major cities in the mid-west of Mauritius (Quatre Bones and Vacoas) and were first assessed at the age of 3 years in 1972. Attrition was generally low, and dropouts were not found to differ from tested participants for various factors, such as gender, ethnicity, cognitive functioning, body size, or psychosocial variables [20]. The ethnic background of the sample was representative of the general population of Mauritius, the majority being of Asian, African, or Middle Eastern origin. The present analyses comprise data from three waves: when participants were 3 years (*M =* 3.06, *SD =* 0.14), 11 years (*M =* 11.04, *SD =* 0.71), and 17 years old (*M =* 17.20, *SD = 0*.75).

The birth cohort of 1,795 children was first assessed at age 3 years on a variety of measures, including indicators of psychosocial adversity, malnutrition, and cognitive functioning. At ages 11 and 17 years, participants’ cognitive functioning was reassessed, with data being available for 1,271 and 823 participants, respectively. During assessment at age 11 years, a major cyclone destroyed parts of the research facilities and brought testing to a halt after approximately two thirds of the sample had been assessed, whereas attrition at age 17 years was mainly due to lack of financial resources [20]. However, previous studies showed that there were no differences between those who were tested and those who were not on various indicators, including gender, socio-demographic factors, IQ, temperament, autonomic functioning, and body size [20].

Verbal informed consent was obtained from participants’ caregivers in accordance with the principles outlined in the hitherto prevailing first version of the Declaration of Helsinki [22]. In addition, written informed consent was obtained from the participants at age 17 years in accordance with the principles outlined in the Belmont report [23]. Data used in the current study was made available by Peter Venables, initiator the Mauritius Child Health Project. Institutional review board approval for secondary data analyses was obtained from the University of Innsbruck.

### Early childhood poverty factors

Because *early childhood poverty factors* largely coincide with *early childhood risk factors* in the context of the present study, we use the two terms interchangeably. Furthermore, as both can be considered a form of adversity, the term *adversity* is also used to denote poverty-related risk factors to which the children in our sample were exposed. In the present sample, both psychosocial and biological risk factors were assessed. Indicators of psychosocial adversity were related to parental characteristics and home resources, comprising maternal and paternal occupational status (coded on an 8-point scale, see S1 Table in S1 File for details), mother’s and father’s years of education, number of people per room living in the house, and general rating of the condition of the house (i.e., *poor*–*average*–*good*). Data on these adversity components were gathered by social workers who visited children’s homes and interviewed caregivers when children were 3 years old.

Biological risk factors were assessed at age 3 years and comprised indicators of stunting and anemia. Stunting, that is, children being too small for their age, was derived from the observed height as a percentage of the expected height for age 3 years (for details see [24]) and is indicative of chronic malnutrition. Expected heights were calculated separately for girls and boys of Indian and African descent to account for relative group differences in height. These adjustments are in line with findings that indicate that ethnic-specific child growth references allow for more valid estimates particularly in Asian populations [25]. For the final stunting measure, percentages were *z*-transformed and converted, with positive values indicating greater stunting. Stunting is considered to be one of the major factors of poverty-related cognitive impairments in low- and middle-income countries [4] and was even found to have intergenerational effects [26]. Anemia was measured from the hemoglobin level (g/dl) assessed by laboratory blood analysis. Besides indicating malnutrition, anemia is closely related to the development of infectious diseases, which also might have adverse effects on cognitive development [4].

### Cognitive functioning measures

Throughout the whole project, cognitive functioning of participants was assessed three times at ages 3, 11 and 17 years.

#### Cognitive functioning at age 3

IQ at age 3 years was assessed by using six components of the Boehm Test of Basic Concepts–Preschool Version (BTBC-P; [27]). Children had to make constructions from blocks; copy shapes; identify body parts; differentiate lengths, numbers, and sizes; name and identify colors; and discriminate between same and different objects. Wording and items were modified to better fit the cultural context [28]. Previous analyses of the data set in which exploratory factor analysis was used yielded the expected two-factor structure, representing verbal (four subtests) and performance ability (two subtests) [28]. Cognitive skills assessed were largely parallel to those assessed by the Wechsler Preschool and Primary Scale of Intelligence (WPPSI; [29]).

#### Cognitive functioning at age 11

*Wechsler Intelligence Scale for Children–Revised*. IQ at age 11 years was assessed by using six subtests of the Wechsler Intelligence Scale for Children—Revised (WISC-R; [30]). Verbal IQ was assessed by the subtests Similarities and Digit Span, whereas Picture Completion, Block Design, Object Assembly, Coding, and Mazes formed an estimate for performance IQ. A distinct feature of the Wechsler intelligence tests is comparability across different test versions that allow a continuous assessment and comparison of cognitive functioning form early childhood to late adulthood [31].

*Trail Making Test*. The Trail Making Test (TMT) was used to assess cognitive performance related to visual search, perceptuo-motor coordination, serial strategy and working memory [32, 33]. It comprised two subtests, in which participants had to connect digits from 1 to 9 (Form A) or alternating digits and letters 1-A to 9-J (Form B) in serial order. The test was administered using a paper&pencil format. Scores reflect the time taken for each subtest.

#### Cognitive functioning at age 17

Cognitive tests at age 17 years were taken from the computerized Automated Psychological Test System by Levander and Elithorn [34], a battery that shares features with the nowadays widely used Cambridge Neuropsychological Test Automated Battery. All tests were implemented on an Apple II computer and conducted at laboratory facilities.

*Trail Making Test*. Similar to age 11 years, performance on the TMT was assessed. At age 17 years, however, a computerized test version was administered, with the cursor being controlled by a joystick and characters being presented on a computer monitor. Participants were instructed to hit each character in serial order as fast as possible. Both subtests were administered twice, once with the preferred hand and once with the non-preferred hand and were preceded by a training session each. Scores are the means of individual hit times for both subtests for the preferred and non-preferred hand.

**Fig 1:**
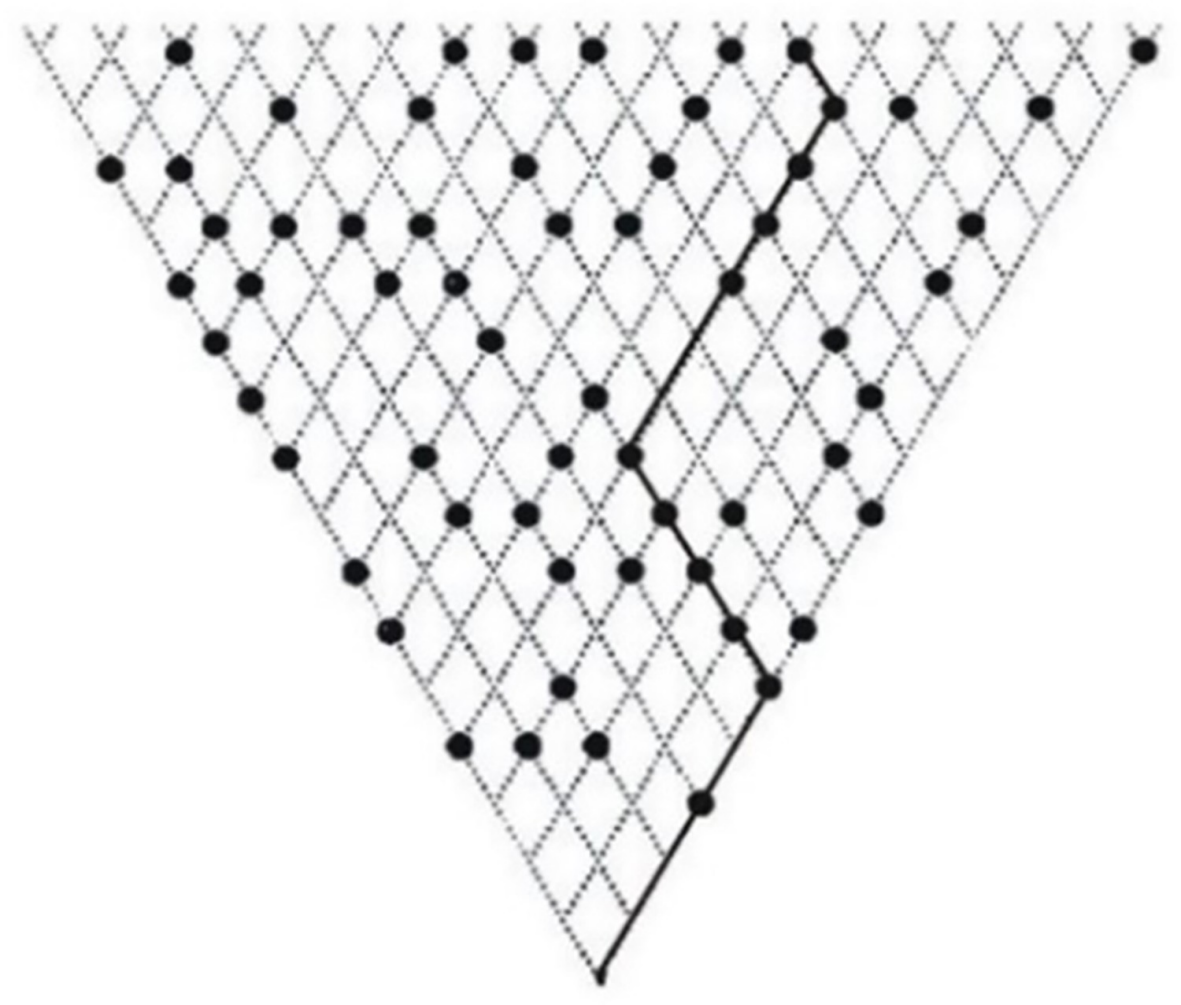
Sample item of the Perceptual Maze Test.

*Perceptual Maze Test*. The Perceptual Maze Test (PMT; [35]) was used to assess visuo-spatial skills, in particular visual scanning. It also acted as an indicator for general intelligence given the sizeable associations between *g* and performance on the Maze test [34, 36]. Participants were presented with triangular lattices that consisted of dotted lines with target dots at the nodes (Fig 1). Within a timeframe of 25 s for each maze, subjects had to find a pathway that connected the largest number of target dots starting from the bottom. Pathways were drawn by using the left and right arrow keys. Further keys allowed participants to erase the last move or save the current pathway as a final solution. If the solution was correct, a new maze with one additional row was presented. If the solution was incorrect or if the time limit expired before a final solution was saved, a new maze with one row less was presented. If participants were unsuccessful two times in a row, the next maze presented had two rows less. After instruction, participants could practice on four-row examples until the task was fully understood. There were 20 trials in total. Scores assessed include the largest maze that subjects were able to solve correctly, processing speed, and inspection speed.

### Data analysis

Due to a high proportion of missing data for the follow-up assessments, missing information was estimated using multivariate imputation by chained equations (MICE). Multiple imputation has repeatedly been demonstrated to outperform other means of dealing with missing data, especially in large samples or repeated measurement designs [37, 38]). Also, the fact that multiple imputation has been found to give less biased results for both missing not at random (MNAR) and missing at random (MAR) data and higher power for missing completely at random (MCAR) data compared to listwise deletion makes it a powerful tool for dealing with missing data [39]. Recent simulation studies showed that well-specified imputation models provide unbiased results even with up to 90% of missing data [40].

One of the many advantages of multiple imputation is the possibility to include auxiliary variables in the imputation model to improve the estimation of missing values. These variables are not part of the analysis model but are either highly correlated to those included or help to explain the mechanisms of missingness [37]. For the imputation model, thus, only auxiliary variables that were correlated on a scale of ±.40 or greater with variables that were to be imputed were chosen, following suggestions by Hardt et al. [37].

In order to obtain an overall measure of cognitive functioning for each time point, cognitive indicator variables were standardized and then averaged. To account for relations between the raw indicators, their standardized counterparts as well as the composite scores, passive imputation was deployed. Passive imputation allows for imputing related variables by sustaining consistency among the different imputations of the same data set [41]. In order to prevent any feedback loops, the predictor matrix was adapted so that the standardized variables as well as the composite indicators were not used as predictors in the imputation model (cf. [42]). Moreover, minimum and maximum values for ordinal variables indicating maternal and paternal occupational status as well as condition of the house were set in post-processing (cf. [41]). To determine the number of imputations needed, the two-stage procedure by von Hippel was applied using the R package *how_many_imputations* [43]. This procedure allows to obtain replicable standard error estimates [43]. A total of 186 imputation was estimated, which consequently was used as a basis for the final imputation algorithm.

Upon imputing missing values, in a first step, we examined the structure of the cognitive functioning indicators by means of confirmatory factor analysis. We report comparative fit index (CFI), Tucker-Lewis Index (TLI), and incremental fit index (IFI). Values of .90 and above are generally taken to indicate acceptable fit and values of .95 and above to indicate good fit. In addition, root mean square error of approximation (RMSEA) and standardized root mean square residual (SRMR) are reported. For both indexes, we adopted the criteria of .08 and below as indicating acceptable fit and values of .05 and below as indicating good fit for RMSEA [44, 45]. To compare the fit of non-nested models, we analyzed differences in Bayesian information criterion values [46]. In a second step, we examined associations between early childhood risk factors and cognitive functioning over time, using hierarchical regression models. Both standardized and non-standardized estimates are reported.

In order to examine change over time, we also tested a latent growth model with early childhood risk factors and sex as predictors of initial and change levels of cognitive functioning. However, as cognitive measures somewhat varied across the three assessments, thus limiting the validity of the model, we decided to report more robust hierarchical regressions in the present article instead. Results of the latent growth model analysis were found to be similar to those of the hierarchical regression analyses. All additional analyses results are available in S2 File.

All analyses were conducted in *R* (v3.13.0; [47]) and codes are available on request from the authors.

## Results

### Missing data analysis

**Fig 2:**
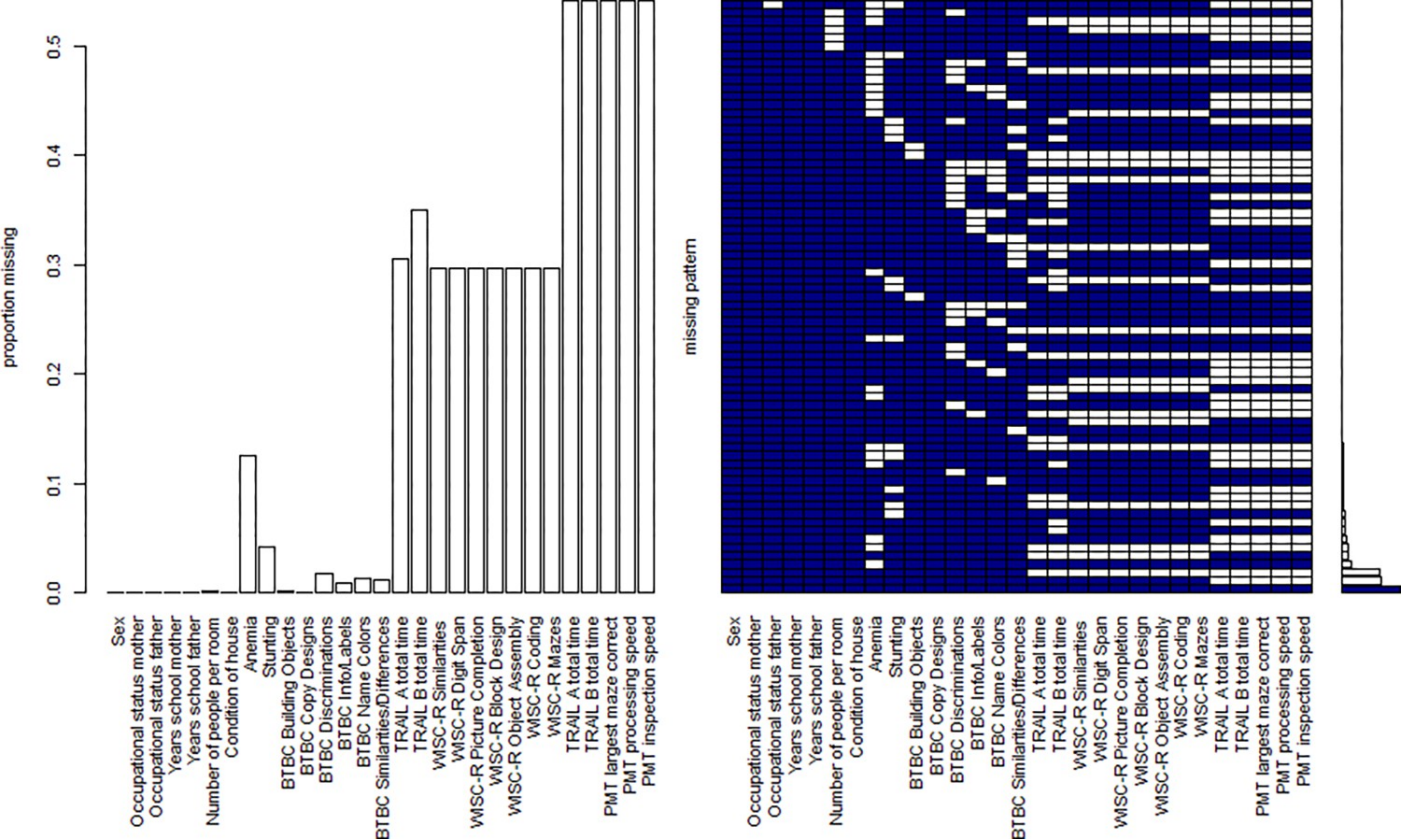
Missing data pattern for variables included in the analysis. The bar chart on the left depicts the proportion of data missing for each variable. The graph on the right displays the different data patterns, with missing data in white and available data in blue. The histogram shows the frequency of the individual data patterns, with complete cases accounting for 28.0% of the total sample.

Out of 1795 cases, 72.0% were missing at least one data point. Missingness of cognitive performance indicators was 0.00% to 1.8% for age 3, 29.6% to 33.12% for age 11, and 54.2% for age 17 years. An overview on missing data patterns can be found in Fig 2. Occupational status of father and mother were slightly higher for cases with missing values, with *Χ^2^*(8) = 27.35, *p* < .001, *ω* = 0.12 and *Χ^2^*(7) = 16.43, *p* = .025, *ω* = 0.10, respectively. Also, participants with missing values showed slightly better performance on the Trail Making Test B (*t*(812.83) = -1.87, *p* = .062) and the WISC-R subtest Digit Span (*t*(870.57) = -1.99, *p* = .047), both assessed at age 11 years. Other than that, no significant differences in socio-demographic variables or cognitive performance at ages 3, 11 and 17 years were found (cf. S1 and S2 Tables in S1 File).

Given the marginal differences between participants with and without missing data and the fact that the majority of missing cases was caused by external factors, with the assessment at age 11 years being brought to a halt after a cyclone had destroyed the laboratory and the assessment at age 17 years being stopped due to a lack in funding, it is assumed that data is at least missing at random. Little’s MCAR test indicated that data is not missing completely at random for the subset of variables included in the analyses, with *Χ^2^*(1462) = 1769.50, *p* < .001, thus violating the assumption for listwise deletion. Consequently, multiple imputation was deployed to handle missing values. Analyses, however, were also performed with a sample consisting of complete cases only for comparison, with results being quite similar to those of the imputed data set. Analyses are available upon request from the authors.

### Descriptive statistics

S3 Table in S1 File summarizes the descriptive statistics of psychosocial adversity indicators. As the sample comprised mainly low- and middle-class families, many parents had little to no education. On average, fathers attended school longer (*M* = 4.55 years, *SD* = 3.58) than mothers did (*M =* 3.98 years, *SD* = 3.25), with *t*(3585.64) = 4.98, *p* < .001, tended to have jobs of higher occupational status (*Mdn = 3*) than mothers (*Mdn =* 1), with the scale ranging from 1 to 8 and *U* = 461321.00, *p* < .001, and often were single earners, as most women (73.1%) did not have a paid job at all compared to only 3.2% of men that were unemployed. More than three quarters of the children grew up in overcrowded homes, with a mean of 3.93 (*SD* = 1.79) people sharing a room. There were no sex differences in risk factors at age 3 years, except for boys experiencing lower levels of stunting, with *t*(1686.37) = -4.58, *p <* .001 (cf. S3 Table in S1 File).

### Confirmatory factor analysis

Measurement models for cognitive functioning at ages 3, 11, and 17 years were tested by using confirmatory factor analysis. Descriptive statistics for cognitive functioning indicators can be found in S3 Table, zero-order correlations are reported in S4 Table (both S1 File). For all ages we tested an one-factor model in order to represent an overall cognitive functioning composite. Moreover, for ages 11 and 17 years we additionally tested a two-factor model to account for the fact that two different measures were used at each assessment. At last, as previous analyses by Raine et al. [28] suggested a two-factor structure for standardized intelligence tests used, we additionally tested a two-factor model at age 3 years and a three-factor model at age 11 years in order to account for a verbal and a performance component for both the BTBC-P and the WISC-R. Fit indices for the models are reported in Table 1. Standardized and unstandardized coefficients for the models are reported in S5 Table in S1 File.

**Table 1:** Fit indices for confirmatory factor analyses.

Model	χ^2^ (df)	CFI	TLI	IFI	RMSEA	90% CI	SRMR	Δ*χ^2^*
**Age 3 years**								
one factor	110.38** (9)	.975	.958	.975	.079**	[.066;.093]	.028	ref.
two factors	94.26** (8)	.978	.959	.978	.078**	[.064;.092]	.026	16.12**
**Age 11 years**								
one factor	288.18** (27)	.915	.886	.915	.073**	[.066;.081]	.048	ref.
two factors	288.19** (26)	.914	.881	.915	.075**	[.067;.083]	.048	0.01 *ns*
three factors	264.04** (24)	.922	.882	.922	.075**	[.067;.083]	.046	24.15**
**Age 17 years**								
one factor	436.48** (5)	.740	.479	.740	.219**	[.202;.237]	.130	ref.
two factors	34.76** (4)	.981	.954	.982	.065**	[.046;.086]	.030	402.27**

CFI, comparative fit index; TLI, Tucker-Lewis index; IFI, incremental fit index. RMSEA, root mean square error of approximation; 90% CI, 90% confidence interval for population RMSE; SRMR, standardized root mean square residual; Δ*χ^2^*, change in chi-square; ref., reference.

*ns* = non-significant.

**p < .01.

Overall, all cognitive functioning models showed acceptable to good fit, except for the one-factor model for age 17 years (cf. Table 1). Also, for models of cognitive functioning age 11 years, TLI values were falling slightly below cut-off limits whereas all other fit indicators were within the bounds of acceptability. As expected, models comprising a verbal and a performance component of standardized intelligence tests at ages 3 and 11 years fit significantly better than a one-factor model, yet differences were only marginal. Also, models comprising an individual factor for each test used showed a slightly better fit than one-factor models. However, as the general one-factor model provided a more favorable balance between factorial validity, parsimony, and comparability across assessment waves and also showed satisfactory fit, we retained the one-factor model for ages 3 and 11 years. For age 17 years the fit of the one-factor model was not satisfactory, thus the subsequent analyses will be conducted for performance on the Tail Making Test and on the Perceptual Maze Test separately. For selected analyses, we have additionally included an overall measure of cognitive functioning at age 17 years in order to allow for comparability across assessment waves. Yet results need to be interpreted with caution as the one-factor model showed a low fit. For the subsequent analyses, indicators were standardized and composites were computed as their respective means.

### Predictors of cognitive functioning

Overall, cognitive functioning was positively intercorrelated across all three time points (Table 2). In line with previous findings (e.g., [1]), the size of associations increased with age and was stronger for more proximate assessments.

**Table 2:** Zero-order correlations and internal consistency estimates (*ω*) for cognitive functioning composites for ages 3, 11, and 17 years for the imputed data.

	*M*	*SD*	1	2	3	4	5	6	7
** One-factor composites**									
1. Cognitive functioning age 3 years	-0.01	0.76	(.90)						
2. Cognitive functioning age 11 years	0.00	0.63	.32	(.89)					
3. Cognitive functioning age 17 years	0.00	0.70	.17	.60	(.90)				
**Two-factor composites**									
4. WISC-R (age 11 years)	0.00	0.73	.34	.97	.60	(.87)			
5. TMT 11 (age 11 years)	0.00	0.74	.11	.60	.31	.39	(.87)		
6. PMT (age 17 years)	0.00	0.85	.14	.48	.89	.49	.22	(.85)	
7. TRTMTAIL 17 (age 17 years)	0.00	0.93	.16	.55	.79	.54	.32	.44	(.85)

*N =* 1795. Positive values for mean composites indicate higher cognitive performance. The one-factor composite for age 3 years is equal to the general intelligence estimate of the Boehm Test of Basic Concepts–Preschool Version (BTBC-P). PMT, Perceptual Maze Test; TMT 11, Trail Making Test assessed at age 11 years; TMT 17, Trail Making Test assessed at age 17 years; WISC-R, Wechsler Intelligence Scale for Children–Revised.

All *p*s < .01.

To examine the relative influence of poverty-related risk factors on cognitive functioning, we run separate hierarchical linear regressions for ages 3, 11 and 17 years. In a first step, individual risk factors assessed at age 3 years were entered. In a next step, previously assessed cognitive functioning indicators were added for ages 11 and 17 years. Then sex was added to all models. In a last step, interactions between risk factors as well as previously assessed cognitive functioning indicators and sex were included. Estimates for the last step are reported in S6 Table in S1 File, whereas estimates for steps one to three are reported in Tables 3–6. Early childhood risk factors were found to predict cognitive performance at ages 3 and 11 years, but not at age 17 years. For age 3 years, risk factors alone explained about 9% of variance (cf. Table 3), while for age 11 years almost a fourth of variance was explained (cf. Table 4). In both cases, adding further predictors, such as sex or previous cognitive performance, did only marginally improve the amount of variance explained. For age 17 years, early childhood risk factors were found to account for about 5% of variance, while adding indicators of cognitive performance at ages 3 and 11 years increased the amount of variance explained considerably to about one-third (cf. Tables 5 and 6). Interaction-terms between sex and the other predictors were found to have only a minimal effect on the variance of each model (cf. S6 Table in S1 File).

**Table 3:** Regression results for cognitive functioning at age 3 years predicted by early childhood risk factors (age 3 years) and sex.

	Model 1			Model 2		
Predictor	*b*	*SE*	*β*	*b*	*SE*	*β*
(Intercept)	-0.33**	0.08		-0.37**	0.08	
Years school father	0.02**	0.01	0.10	0.02**	0.01	0.10
Years school mother	0.01	0.01	0.04	0.01	0.01	0.04
Occupational status father	0.02*	0.01	0.06	0.03*	0.01	0.06
Occupational status mother	0.05**	0.01	0.10	0.05**	0.01	0.09
Number of people per room	-0.02	0.01	-0.03	-0.02	0.01	-0.03
Condition of house	0.02	0.03	0.02	0.02	0.03	0.02
Anaemia	-0.06**	0.01	-0.11	-0.06**	0.01	-0.11
Stunting	-0.07**	0.02	-0.09	-0.07**	0.02	-0.10
Sex:female				0.09*	0.03	0.05
Fit	*R*^*2*^ = .088 | *R*^2^_adj_ = .084			*R*^*2*^ = .091 | *R*^2^_adj_ = .087		
F for change in R^2^				6.194*		

*N* = 1795. Model 1 = Risk factors, Model 2 = Risk factors + sex, Model 3 = Risk factors + sex + risk factors*sex interactions.

+ *p* < .10.

** *p* < .01.

* *p* < .05.

**Table 4:** Regression results for cognitive functioning at age 11 years predicted by early childhood risk factors (age 3 years) and cognitive functioning at age 3 years as well as sex.

	Model 1			Model 2			Model 3		
Predictor	*b*	*SE*	*β*	*b*	*SE*	*β*	*b*	*SE*	*β*
(Intercept)	-0.46**	0.07		-0.41**	0.07		-0.33**	0.07	
Years school father	0.02**	0.01	0.09	0.01*	0.01	0.07	0.01*	0.01	0.07
Years school mother	0.03**	0.01	0.15	0.03**	0.01	0.14	0.03**	0.01	0.14
Occupational status father	0.04**	0.01	0.11	0.03**	0.01	0.10	0.03**	0.01	0.09
Occupational status mother	0.04**	0.01	0.09	0.03**	0.01	0.07	0.03**	0.01	0.07
Number of people per room	-0.03**	0.01	-0.08	-0.02**	0.01	-0.07	-0.02**	0.01	-0.07
Condition of house	0.10**	0.03	0.08	0.10**	0.03	0.08	0.10**	0.03	0.08
Anaemia	-0.04**	0.01	-0.07	-0.03*	0.01	-0.05	-0.03*	0.01	-0.05
Stunting	-0.13**	0.02	-0.19	-0.12**	0.02	-0.18	-0.11**	0.02	-0.16
BTBC-P				0.17**	0.02	0.20	0.17**	0.02	0.21
Sex:female							-0.17**	0.03	-0.14
Fit	*R^2^* = .234 | *R^2^*_*adj*_ = .230			*R^2^* = .271 | *R^2^*_*adj*_ = .270			*R^2^* = .288 | *R^2^*_*adj*_ = .284		
F for change in R^2^				70.375**			32.027**		

*N* = 1795. BTBC-P, Boehm Test of Basic Concepts–Preschool Version. Model 1 = Risk factors, Model 2 = Risk factors + cognitive functioning (age 3 years), Model 3 = Risk factors + cognitive functioning (age 3 years) + sex, Model 4 = Risk factors + cognitive functioning (age 3 years) + sex + risk factors*sex interactions + cognitive functioning (age 3 years)*sex interaction.

+ *p* < .10.

** *p* < .01.

* *p* < .05.

**Table 5:** Regression results for performance on the Trail Making Test at age 17 years predicted by early childhood risk factors (age 3 years) as well as cognitive functioning at ages 3 and 11 years and sex.

	Model 1			Model 2			Model 3			Model 4		
Predictor	*b*	*SE*	*β*	*b*	*SE*	*β*	*b*	*SE*	*β*	*b*	*SE*	*β*
(Intercept)	-0.30*	0.13		-0.25+	0.13		0.10	0.12		0.12	0.13	
Years school father	0.02+	0.01	0.08	0.02+	0.01	0.07	0.01	0.01	0.02	0.01	0.01	0.02
Years school mother	0.01	0.01	0.02	0.00	0.01	0.02	-0.02+	0.01	-0.06	-0.02+	0.01	-0.06
Occupational status father	0.00	0.02	0.01	0.00	0.02	0.00	-0.03	0.02	-0.05	-0.03	0.02	-0.05
Occupational status mother	0.03	0.02	0.04	0.02	0.02	0.03	-0.01	0.02	-0.01	0.00	0.02	-0.01
Number of people per room	-0.01	0.02	-0.01	0.00	0.02	-0.01	0.02	0.01	0.03	0.02	0.01	0.03
Condition of house	0.12*	0.06	0.07	0.12*	0.06	0.07	0.04	0.05	0.02	0.04	0.05	0.02
Anaemia	-0.01	0.02	-0.02	0.00	0.02	0.00	0.03	0.02	0.03	0.03	0.02	0.03
Stunting	-0.13**	0.03	-0.13	-0.12*	0.03	-0.12	-0.01	0.03	-0.01	-0.01	0.03	-0.01
BTBC-P				0.14*	0.04	0.11	-0.01	0.04	-0.01	0.00	0.04	-0.01
TMT 11							0.17*	0.05	0.14	0.17*	0.05	0.14
WISC-R							0.69*	0.05	0.52	0.68*	0.05	0.51
Sex:female										-0.05	0.06	-0.03
Fit	*R*^*2*^ = .053 | *R^2^*_*adj*_ = .048			*R*^*2*^ = .065 | *R^2^*_*adj*_ = .060			*R*^*2*^ = .315 | *R^2^*_*adj*_ = .310			*R*^*2*^ = .316 | *R^2^*_*adj*_ = .311		
F for change in R^2^				12.559**			119.511**			0.884		

*N* = 1795. BTBC-P, Boehm Test of Basic Concepts–Preschool Version; TMT 11, Trail Making Test assessed at age 11 years; WISC-R, Wechsler Intelligence Scale for Children–Revised. Model 1 = Risk factors, Model 2 = Risk factors + cognitive functioning (age 3 years), Model 3 = Risk factors + cognitive functioning (ages 3 and 11 years), Model 4 = Risk factors + cognitive functioning (ages 3 and 11 years) + sex, Model 5 = Risk factors + cognitive functioning (ages 3 and 11 years) + sex + risk factors* sex interactions + cognitive functioning (ages 3 and 11 years)*sex interactions.

** *p* < .01.

* *p* < .05. + *p* < .10.

**Table 6:** Regression results for performance on the Perceptual Maze Test at age 17 years predicted by early childhood risk factors (age 3 years) as well as cognitive functioning at ages 3 and 11 years and sex based on imputed data.

	Model 1			Model 2			Model 3			Model 4		
Predictor	*b*	*SE*	*β*	*b*	*SE*	*β*	*b*	*SE*	*β*	*b*	*SE*	*β*
(Intercept)	-0.18	0.12		-0.15	0.12		0.15	0.11		0.35*	0.11	
Years school father	0.00	0.01	0.02	0.00	0.01	0.01	-0.01	0.01	-0.04	-0.01	0.01	-0.04
Years school mother	0.00	0.01	0.02	0.00	0.01	0.01	-0.02	0.01	-0.06	-0.01	0.01	-0.05
Occupational status father	0.03	0.02	0.06	0.03	0.02	0.06	0.01	0.02	0.01	0.00	0.02	0.01
Occupational status mother	0.01	0.02	0.02	0.01	0.02	0.01	-0.01	0.02	-0.02	-0.01	0.02	-0.01
Number of people per room	-0.02+	0.01	-0.05	-0.02	0.01	-0.05	-0.01	0.01	-0.02	-0.01	0.01	-0.02
Condition of house	0.06	0.05	0.04	0.06	0.05	0.04	-0.01	0.05	0.00	0.01	0.04	0.01
Anaemia	-0.03	0.02	-0.04	-0.02	0.02	-0.03	0.00	0.02	0.00	0.00	0.02	0.00
Stunting	-0.11**	0.03	-0.12	-0.10**	0.03	-0.11	-0.02	0.03	-0.02	0.00	0.03	0.00
BTBC-P				0.10**	0.04	0.09	-0.03	0.03	-0.02	0.00	0.03	0.00
TMT 11							0.04	0.05	0.04	0.04	0.05	0.03
WISC-R							0.61**	0.05	0.51	0.55**	0.05	0.46
Sex:female										-0.47**	0.05	-0.28
Fit	*R*^*2*^ = .04 | *R^2^*_*adj*_ = .045			*R*^*2*^ = .056 | *R^2^*_*adj*_ = .052			*R*^*2*^ = .255 | *R^2^*_*adj*_ = .250			*R*^*2*^ = .331 | *R^2^*_*adj*_ = .326		
F for change in R^2^				7.623**			81.712**			107.026**		

*N* = 1795. BTBC-P, Boehm Test of Basic Concepts–Preschool Version; PMT, Perceptual Maze Test; TMT 11, Trail Making Test assessed at age 11 years; TMT 17, Trail Making Test assessed at age 17 years; WISC-R, Wechsler Intelligence Scale for Children–Revised. Model 1 = Risk factors, Model 2 = Risk factors + cognitive functioning (age 3 years), Model 3 = Risk factors + cognitive functioning (ages 3 and 11 years), Model 4 = Risk factors + cognitive functioning (ages 3 and 11 years) + sex, Model 5 = Risk factors + cognitive functioning (ages 3 and 11 years) + sex + risk factors*sex interactions + cognitive functioning (ages 3 and 11 years)*sex interactions.

** *p* < .01.

* *p* < .05. + *p* < .10.

Chronic malnutrition as well as parental attainments were found to be the best predictors of initial levels of cognitive functioning (cf. Table 3). Children were found to score lower on the cognitive assessment at age 3 years if they showed signs of stunting (*β =* -0.09) or anemia (*β* = -0.11) and higher if their parents had a higher occupational status (*β* = 0.06 for the father and *β* = 0.09 for the mother). Overall, girls were found to show slightly better cognitive performance at age 3 years than boys, with sex being a significant predictor even when entered after socio-economic risk factors (*β* = 0.05). However, this effect vanished when interactions between early childhood risk factors and sex were included in the regression model. Instead, a significant interaction between paternal educational achievement and sex was found (*β* = 0.07), indicating that the positive association between paternal educational level and cognitive performance was slightly stronger for girls than for boys. Regarding interaction terms, the interaction between sex and paternal educational attainment was found to be a significant, though small-sized predictor (*β* = 0.03, *p* = .015), favoring girls with well-educated fathers (cf. S7 Table in S1 File).

While the initial level of cognitive functioning was the best predictor of cognitive performance at age 11 years (*β* = .21), early childhood risk factors and sex still explained a significant amount of additional variance. In particular, children were found to score higher on cognitive assessments at age 11 years when they had well-educated mothers (*β* = 0.14) and to score lower when experiencing stunting at an early age (*β* = -0.16). Other indicators of malnutrition, parental attainment, and living conditions were also found to show significant but yet weaker associations, with *β*s ranging from -0.07 to 0.09, favoring children experiencing less adversity. Moreover, boys were found to show better performance than girls independent from initial levels of cognitive functioning and risk factors experienced (*β* = -0.14). All predictors combined explained a total variance of 29% (cf. Table 4). No significant interactions between early childhood risk factors and sex were found.

For age 17 years, early childhood risk factors showed no significant association with neither the Trail Making Test performance (cf. Table 5) nor the Perceptual Maze Test performance (cf. Table 6). Also, as with age 11 years, there were no significant interactions between risk factors and sex. However, children scoring higher on cognitive assessment at age 11 years showed better test performance at age 17 years. Performance on the Trail Making Test at age 17 years was best predicted by WISC-R performance (*β* = 0.51), followed by Trail Making Test performance assessed at age 11 years (*β* = 0.14). Moreover, there was a trend of a slightly negative association with maternal academic achievement (*β* = -0.06, *p* = .077). Performance on the Perceptual Maze Test was only associated with performance on the WISC-R (*β* = 0.46), but not the Trail Making Test assessed at age 11 years. Moreover, it was significantly predicted by sex (*β* = -0.28) as well as the interaction between sex and WISC-R performance (*β* = -0.10), with boys showing better Perceptual Maze Test performance in general and also stronger positive associations between WISC-R and Perceptual Maze Test performance. Adding sex as a predictor resulted in 10% of additional variance explained, which accounted for one-third of total variance.

**Fig 3:**
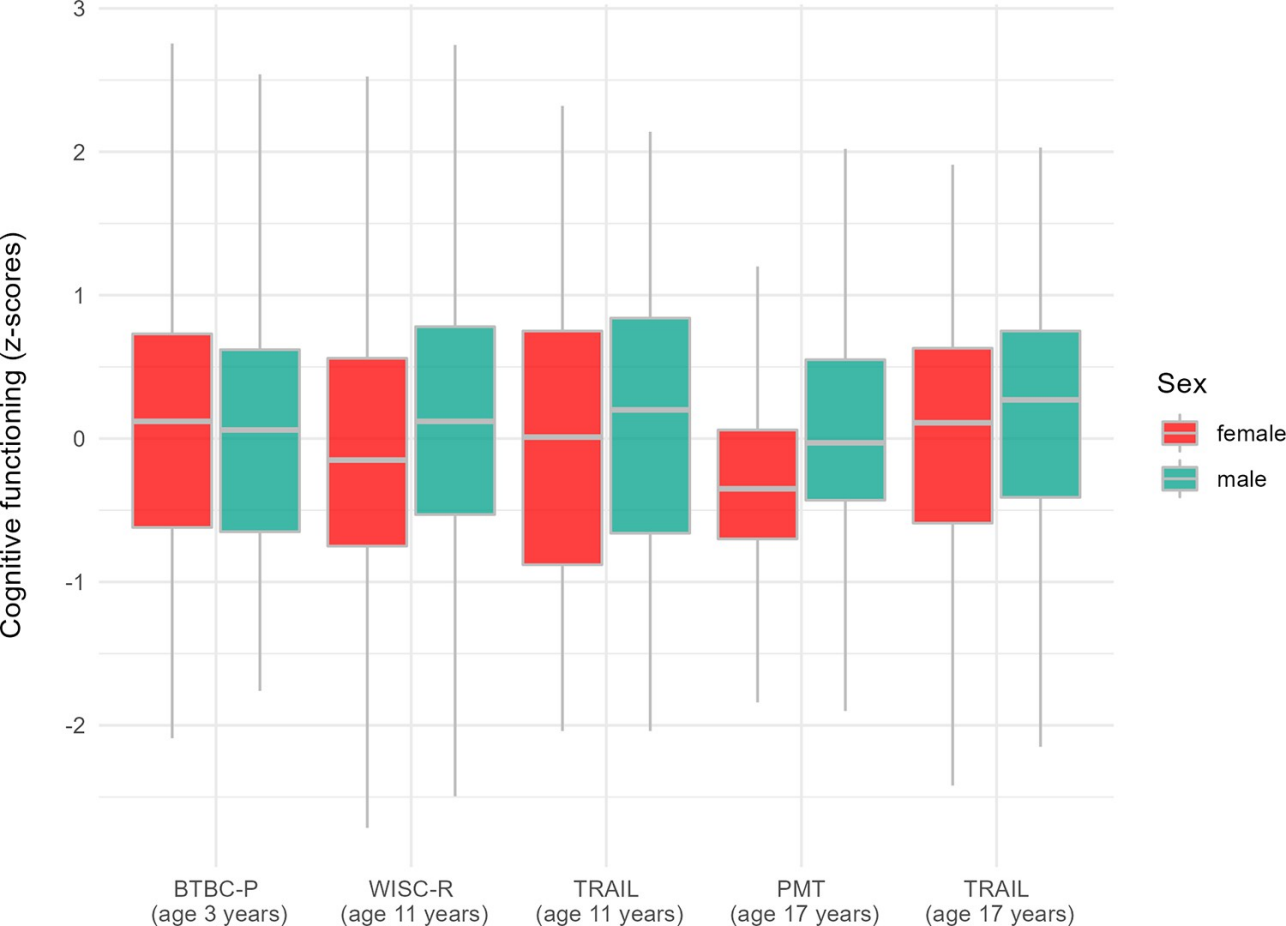
Summary of cognitive functioning indicators for males and females at ages 3, 11, and 17 years based on imputed data.

Overall, the influence of both early childhood risk factors and sex on cognitive functioning was found to vary over time. While sex was only remotely related to initial mean levels, favoring girls, this association reversed throughout childhood and adolescence. Hence, regardless of poverty-related risks, boys’ cognitive functioning improved between age 3 and 17 years, whereas for girls, it deteriorated markedly (cf. Fig 3). Regarding early childhood risk factors, malnutrition and parental attainment proved to be the best predictors of cognitive functioning at ages 3 and 11 years, while risk factors were only marginally associated with cognitive performance at age 17 years. Instead, cognitive functioning in late adolescence was best predicted by cognitive performance at age 11 years and sex.

## Discussion

Using data from a culturally diverse prospective longitudinal study, we examined the influence of early childhood poverty experience on cognitive outcomes at ages 3, 11 and 17 years. Our findings add to previous research about the effects of early childhood poverty on cognitive functioning by drawing on a sample of children who faced a degree of poverty exceeding those studied in most Western samples to date. This feature offers a basis for comparison with what is known about antecedents of cognitive functioning from Western samples.

The evidence that can be used for comparisons is somewhat limited by the scarcity of prospective longitudinal studies that have traced cognitive performance from toddlerhood to late adolescence by using standardized tests. Probably the closest comparison that can be drawn is to the Twins Early Development Study (TEDS), which assessed the intelligence of twins born in England and Wales in the 1990s multiple times from age 2 to 16 years [1]. Using a proxy IQ derived from parental reports at age 2 years and Raven’s progressive matrices and other standard intelligence measures at later ages, von Stumm & Plomin [1] reported that the stability of cognitive ability across the 14-year interval was *r* = .21, which is similar to the *r* = .17 association found across the 14-year span in the current sample. Stability between age 3 years and 10 years was *r* = .31 and from 10 years to 16 years was *r* = .50 in the UK study. In the current sample, intercorrelations between cognitive measures at age 3 years and 11 years and at age 11 years and 17 years were *r* = .32 and *r* = .60, respectively. Despite differences in historical, sociocultural, and socioeconomic contexts, and in the measures used, these studies suggest a strikingly similar increase in rank-order stability of cognitive capabilities over time. The resemblance suggests that the present battery of cognitive tests used in late adolescence and the measures of general intelligence used in the TEDS may tap into similar constructs.

Our results further suggest that socioeconomic deprivation and chronic malnutrition not only impair early childhood cognitive functioning, but they also predict a deterioration in cognitive outcomes over time. This is a pattern known from studies carried out in Western samples [1, 48]. However, unlike previous studies (e.g., [2, 11]), we were able to pinpoint the extent to which variation in individual risk factors affected cognitive outcomes by using continuous assessment rather than a categorical approach. Specifically, chronic malnutrition and parental characteristics were found to show similar-sized, independent associations with cognitive functioning in pre-school (age 3 years) and school age (age 11 years). The size of the associations between parental characteristics and cognitive functioning thereby was relatively similar to those reported in the TEDS [1]. Our findings on the pivotal role of stunting also extend previous analyses of the MCHP, which found that children who showed multiple signs of malnutrition in toddlerhood also showed deceased IQ levels, reading ability, and school performance at age 11 years, independent of the level of psychosocial risks encountered in early childhood [49]. While the global prevalence of stunting has been declining over the last two decades, in 2020, stunting was still affecting more than one in five children under 5 years worldwide and about one in three children under 5 years in Africa and Southern Asia [15], with boys being at particular risk of being undernourished [50]. Numbers like these highlight the importance of tackling malnutrition in order to attenuate its deteriorating effects on cognitive development. Given the promising results of educational, health and nutritional enrichment programs on cognitive development (for an overview see [12]), initiatives like these could help millions of children from low and middle-income countries to live up to their developmental potential [51]. However, implementing initiatives tackling malnutrition and other repercussions of socio-economic disadvantage is not enough. Although we expected sex to play a role in the development of cognitive performance over time because of the gender-unequal social structures and roles prevalent in Mauritius during the period of study, its extent was nevertheless surprising. While girls slightly outperformed boys on cognitive functioning at a young age, this trend dramatically reversed over time. Regardless of poverty risk level experienced in early childhood, boys were found to score substantially higher on cognitive assessments at ages 11 and 17 years. These findings are in line with those by Singh and Krutikova [19], who found that gender gaps in cognitive achievement widened between ages 5 and 15 years in four non-Western countries (Ethiopia, India, Peru, and Vietnam). In contrast, the gender performance gap closed almost entirely in the British TEDS sample throughout adolescence [1]. One likely explanation for these contrasting patterns is that where gender inequality prevails, girls are often denied opportunities for education for a variety of reasons. For example, in the nations studied by Singh and Kurtikova ([19]; i.e. Ethiopia, India, Peru, and Vietnam) boys overall received better schooling and more educational investment compared to girls. Tenuous educational engagement and low educational expectations for girls, combined with prejudices based on gender stereotypes at home, at school, and in the community, thus may partly account for the widening gender gap in cognitive performance over the schooling period in the Mauritian sample and other samples from developing nations.

### Limitations

Results from the current research should be interpreted within its limitations. First, assessments took place during the 1970s and 1980s and the measures used share the limitations of the tests of the time [52]. Although the preschool intelligence measures were culturally adapted, fairly conventional, and more or less comparable to more up-to-date measures (e.g., [36, 53, 54]), the computerized assessment of cognitive abilities at age 17 years was somewhat new. Since Mauritius was a poor country with limited infrastructure at that time [20], it is likely that only a small percentage of participants were familiar with computers or input devices such as joysticks and keyboards, raising concerns about the cultural fairness of the battery. This aspect should be taken into consideration, especially when examining correlations between the Trail Making Test assessed at ages 11 and 17 years, as it may help to explain the unexpectedly low associations over time. However, considering the good fit of the one- and two-factor models of cognitive functioning at ages 11 and 17 years, as well as the sizeable intercorrelations of cognitive indicators, it would seem that participant’s task performance not adversely affected by the then-relatively new technological devices.

Second, cognitive performance was assessed with different measures at ages 3, 11, and 17 years. These differences raise questions about the comparability of constructs across the different assessments. It is a well-known difficulty in longitudinal research that measures taken in early childhood and in adolescence or adulthood cannot be exactly the same due to children’s rapid rate of development and maturation. The important requirement is that changing indicators of cognitive functioning relate to the same construct as children grow older. All cognitive functioning measures used in the current study are either standardized intelligence tests or measures that are highly correlated with estimates of general intelligence [36, 55], hence allowing for an overall comparability across measurement times. Also, the similarity between the present findings and the longitudinal correlations found for intelligence measures in the TEDS study [1], as well as the consistency with effect sizes for SES associations with cognitive attainment reported in other studies (cf. [9]), implies a considerable degree of construct continuity. However in the end, there can be no guarantee, only stronger or weaker evidence for comparability of construct-indicators over time.

Third, the explanations for the associations between sex, adverse environments, and later cognitive outcomes remain speculative. The processes through which poverty impairs cognitive functioning likely involve both social-familial and biological factors. Children from disadvantaged backgrounds may experience less encouragement and opportunity for learning and cognitive engagement than do their better-off peers (for overviews see [12, 56]). Socioeconomic deprivation may also exert its impact via changes in brain structure, particularly in areas related to memory, executive control, and emotion (cf. [57–59]). Also, the design of the study did not allow us to examine genetic effects. Genetic effects on IQ have generally been found to be modest in low-income samples [60, 61], and age-to-age changes in cognitive outcomes appear to be mainly environmentally induced (e.g., [62]). Even so, we cannot rule out that genetic factors may be involved in the association between poverty and later cognitive outcomes.

## Conclusions

This study is one of the few to have systematically and prospectively examined how poverty affects cognitive functioning over time in a then low-income nation. Its findings strongly suggest that cognitive functioning is more adversely affected by poverty in early childhood than it is in Western samples and that the effects are persistent and long-lasting. Despite good progress over the last two decades, rates of stunting in African, Asian and Oceanian regions remain high or even are on the rise [15]. Moreover, children still are more likely to live in poverty than any other age group [63], leaving more than 250 million children in low- and middle-income countries at risk of not reaching their developmental potential [12, 51]. Although initiatives for fighting malnutrition and poverty in low-income nations are crucial, our findings suggest that they may prove ineffectual if they do not at the same time counteract the deleterious effects of gender inequality. In terms of policy recommendation, our results imply that, to prevent cognitive impairment, interventions tackling poverty and malnutrition should focus on the infancy period and be designed in a gender-sensitive way, providing added assistance to girls.

## Supporting information

S1 File Supplemental material. (DOCX)

S2 File Supplemental material on latent growth curve modelling. (DOCX)
