# Supplementary material for: Associations between early childhood poverty and cognitive functioning throughout childhood and adolescence: A 14-year prospective longitudinal analysis of the Mauritius Child Health Project
Source: PLoS One. 2023 Feb 24;18(2):e0278618. doi: 10.1371/journal.pone.0278618 (PMC9956590; doi:10.1371/journal.pone.0278618)
Supplement: S1 File — (DOCX) [file pone.0278618.s001.docx]

**Supplemental Material**

**Table S1. Descriptive Statistics of Demographic and Early Childhood Variables for Participants With and Without Missing Data.**

|  | ***n*** | **% missing in total sample** | **Cases with missing data** | **Cases without any missing data** | ***p*** |
| --- | --- | --- | --- | --- | --- |
|  |  |  | *n* = 1401 | *n* = 394 |  |
| Age Phase A | 1795 | 0.00 | 3.08 (0.15) | 3.05 (0.13) | <.001 |
| Age Phase J | 1260 | 29.81 | 11.10 (0.71) | 11.10 (0.69) | .810 |
| Age Phase M | 697 | 61.17 | 17.30 (0.82) | 17.20 (0.77) | .224 |
| Sex | 1795 | 0.00 |  |  | .237 |
| Male | 930 |  | 715 (51.0%) | 215 (54.6%) |  |
| Female | 865 |  | 686 (49.0%) | 179 (45.4%) |  |
| Occupational status mother | 1795 | 0.00 |  |  | .026 |
| unknown | 206 |  | 152 (11%) | 54 (14%) |  |
| unemployed | 1303 |  | 1008 (72%) | 295 (75%) |  |
| part-time laborer | 7 |  | 7 (0%) | 0 (0%) |  |
| agricultural worker, laborer | 148 |  | 120 (9%) | 28 (7%) |  |
| factory worker, home handicraft worker, cook in a private home | 38 |  | 29 (2%) | 9 (2%) |  |
| salesperson, driver | 33 |  | 28 (2%) | 5 (1%) |  |
| low-level clerk, manager of small business (<20 workers), skilled worker | 6 |  | 6 (0%) | 0 (0%) |  |
| managerial, high-level clerical (20-49 employees), nurse, midwife | 54 |  | 51 (4%) | 3 (1%) |  |
| Occupational status father | 1794 | 0.06 |  |  | .002 |
| unknown | 105 |  | 82 (6%) | 23 (6%) |  |
| unemployed | 57 |  | 47 (3%) | 10 (3%) |  |
| part-time laborer | 37 |  | 20 (1%) | 17 (4%) |  |
| agricultural worker laborer | 761 |  | 578 (41%) | 183 (46%) |  |
| factory worker, home handicraft worker, cook in a private home | 200 |  | 159 (11%) | 41 (10%) |  |
| salesperson driver | 461 |  | 362 (26%) | 99 (25%) |  |
| low-level clerk, manager of small business (<20 workers), skilled worker | 53 |  | 49 (4%) | 4 (1%) |  |
| managerial high-level clerical (20-49 employees) nurse midwife | 114 |  | 99 (7%) | 15 (4%) |  |
| academic manager of moderate or large business (>= 50 employees) | 6 |  | 4 (0%) | 2 (1%) |  |
| Years school mother | 1795 | 0.00 | 4.05 (3.30) | 3.74 (3.04) | .084 |
| Years school father | 1795 | 0.00 | 4.58 (3.62) | 4.44 (3.43) | .493 |
| Number of people per room | 1790 | 0.28 | 3.94 (1.78) | 3.89 (1.83) | .637 |
| Condition of house | 1795 | 0.00 |  |  | .916 |
| poor | 145 |  | 115 (8.21%) | 30 (7.61%) |  |
| average | 1241 |  | 966 (69.0%) | 275 (69.8%) |  |
| good | 409 |  | 320 (22.8%) | 89 (22.6%) |  |
| Anaemia | 1569 | 12.59 | -0.01 (1.37) | 0.02 (1.40) | .753 |
| Stunting | 1720 | 4.18 | 0.00 (1.01) | -0.01 (0.98) | .765 |

*Note.* Median and 95%-CI are reported for continuous variables that are not normally distributed. Absolute and relative frequencies are reported for categorical variables.

**Table S2. Descriptive Statistics of Cognitive Performance Indicators at Ages 3, 11 and 17 Years for Participants With and Without Missing Data.**

|  | ***n*** | **% missing in total sample** | **Cases with missing data** | **Cases without missing data** | ***p*** |
| --- | --- | --- | --- | --- | --- |
|  |  |  | ***n* = 1401** | ***n* = 394** |  |
| **Age 3 years** |  |  |  |  |  |
| BTBC Building Objects | 1791 | 0.22 | 2.67 (1.40) | 2.61 (1.33) | .435 |
| BTBC Copy Designs | 1795 | 0.00 | 1.32 (1.30) | 1.31 (1.31) | .954 |
| BTBC Size/Length/# Discriminations | 1762 | 1.84 | 2.94 (2.19) | 2.87 (2.19) | .561 |
| BTBC Info/Labels | 1777 | 1.00 | 11.90 (7.83) | 12.10 (8.05) | .651 |
| BTBC Name Colors | 1771 | 1.34 | 1.79 (2.58) | 1.58 (2.35) | .134 |
| BTBC Similarities/Differences | 1773 | 1.23 | 1.53 (1.50) | 1.44 (1.49) | .334 |
| **Age 11 years** |  |  |  |  |  |
| TRAIL A mean time | 1264 | 29.58 | 3.84 (2.41) | 3.63 (1.48) | .056 |
| TRAIL B mean time | 1200 | 33.15 | 14.20 (7.66) | 15.00 (7.32) | .062 |
| WISC-R Similarities | 1262 | 29.69 | 8.21 (4.03) | 8.13 (3.64) | .710 |
| WISC-R Digit Span | 1262 | 29.69 | 8.10 (2.08) | 8.33 (1.80) | .047 |
| WISC-R Picture Completion | 1262 | 29.69 | 8.99 (2.83) | 9.05 (2.55) | .749 |
| WISC-R Block Design | 1262 | 29.69 | 16.3 (10.7) | 16.70 (9.72) | .544 |
| WISC-R Object Assembly | 1262 | 29.69 | 13.7 (6.80) | 14.10 (6.58) | .294 |
| WISC-R Coding | 1262 | 29.69 | 34.0 (11.4) | 35.00 (10.2) | .128 |
| WISC-R Mazes | 1262 | 29.69 | 13.90 (5.18) | 14.40 (4.55) | .097 |
| **Age 17 years** |  |  |  |  |  |
| TRAIL A mean time | 823 | 54.15 | 2.26 (0.49) | 2.29 (0.56) | .370 |
| TRAIL B mean time | 823 | 54.15 | 3.05 (0.92) | 3.08 (0.90) | .680 |
| PMT largest maze correct | 823 | 54.15 | 11.20 (2.49) | 10.90 (2.31) | .125 |
| PMT processing speed | 823 | 54.15 | 1.95 (1.02) | 1.89 (0.99) | .343 |
| PMT inspection speed | 823 | 54.15 | 8.78 (10.7) | 8.79 (11.7) | .994 |

*Note.* Median and 95%-CI are reported for continuous variables that are not normally distributed. Absolute and relative frequencies are reported for categorical variables.

**Table S3. Descriptive Summary of Early Childhood Socio-Demographic and Risk Variables as well as Indicators of Cognitive Functioning at Ages 3, 11 and 17 Years for Original and Imputed Data as well as Pooled Mean Differences for Imputed Data by Sex.**

|  |  | **Original data** | | | | |  | **Imputed data** | |  | **Sex differences** | | |
| --- | --- | --- | --- | --- | --- | --- | --- | --- | --- | --- | --- | --- | --- |
|  |  | *n* | *M* | *SD* | *Min* | *Max* |  | *M* | *SD* |  | Mean Difference | *t* | *p* |
| **Demographics & risk factors (age 3 years)** |  |  |  |  |  |  |  |  |  |  |  |  |  |
| Occupational status mother |  | 1795 | 2.39 | 1.40 | 1.00 | 8.00 |  | 2.39 | 1.40 |  | -0.06 | -0.95 | .342 |
| Occupational status father |  | 1794 | 4.73 | 1.63 | 1.00 | 9.00 |  | 4.72 | 1.63 |  | 0.13 | 1.74 | .082 |
| Years school mother |  | 1795 | 3.98 | 3.25 | 0.00 | 16.00 |  | 3.98 | 3.25 |  | 0.10 | 0.63 | .526 |
| Years school father |  | 1795 | 4.55 | 3.58 | 0.00 | 19.00 |  | 4.55 | 3.58 |  | 0.26 | 1.52 | .129 |
| Number of people per room |  | 1790 | 3.93 | 1.79 | 1.00 | 13.00 |  | 3.93 | 1.79 |  | -0.06 | -0.69 | .487 |
| Condition of house |  | 1795 | 1.15 | 0.54 | 0.00 | 2.00 |  | 1.15 | 0.54 |  | 0.00 | 0.11 | .914 |
| Anemia |  | 1569 | 0.00 | 1.38 | -3.71 | 4.99 |  | 0.00 | 1.38 |  | -0.11 | -1.55 | .120 |
| Stunting |  | 1720 | 0.00 | 1.00 | -2.47 | 3.19 |  | 0.00 | 1.00 |  | -0.22 | -4.58 | < .001 |
| **Cognitive functioning (age 3 years)** |  |  |  |  |  |  |  |  |  |  |  |  |  |
| BTBC Building Objects |  | 1791 | 2.65 | 1.38 | 0.00 | 5.00 |  | 2.65 | 1.38 |  | -0.08 | -1.26 | .207 |
| BTBC Copy Designs |  | 1795 | 1.32 | 1.31 | 0.00 | 6.00 |  | 1.32 | 1.31 |  | -0.13 | -2.06 | .040 |
| BTBC Size/Length/# Discriminations |  | 1762 | 2.92 | 2.19 | 0.00 | 14.00 |  | 2.94 | 2.19 |  | 0.07 | 0.63 | .526 |
| BTBC Info/Labels |  | 1777 | 11.98 | 7.88 | 0.00 | 21.00 |  | 11.98 | 7.87 |  | -0.50 | -1.33 | .184 |
| BTBC Name Colors |  | 1771 | 1.74 | 2.53 | 0.00 | 15.00 |  | 1.75 | 2.54 |  | -0.16 | -1.30 | .192 |
| BTBC Similarities/Differences |  | 1773 | 1.51 | 1.50 | 0.00 | 4.00 |  | 1.51 | 1.50 |  | -0.11 | -1.62 | .105 |
| **Cognitive functioning (age 11 years)** |  |  |  |  |  |  |  |  |  |  |  |  |  |
| TRAIL A mean time |  | 1248 | 90.37 | 30.96 | 27.00 | 150.00 |  | 90.65 | 30.98 |  | -4.75 | -2.86 | .004 |
| TRAIL B mean time |  | 1167 | 197.97 | 65.78 | 60.00 | 300.00 |  | 202.34 | 66.65 |  | -5.43 | -1.55 | .120 |
| WISC-R Similarities |  | 1262 | 8.19 | 3.91 | 0.00 | 23.00 |  | 8.22 | 3.87 |  | 0.25 | 1.23 | .219 |
| WISC-R Digit Span |  | 1262 | 8.17 | 2.00 | 0.00 | 15.00 |  | 8.20 | 1.95 |  | 0.06 | 0.64 | .521 |
| WISC-R Picture Completion |  | 1262 | 9.01 | 2.74 | 0.00 | 19.00 |  | 9.04 | 2.71 |  | 0.90 | 6.37 | < .001 |
| WISC-R Block Design |  | 1262 | 16.46 | 10.43 | 0.00 | 48.00 |  | 16.50 | 10.32 |  | 3.05 | 5.70 | < .001 |
| WISC-R Object Assembly |  | 1262 | 13.85 | 6.73 | 0.00 | 44.00 |  | 13.90 | 6.67 |  | 2.34 | 6.68 | < .001 |
| WISC-R Coding |  | 1262 | 34.29 | 11.08 | 0.00 | 71.00 |  | 34.43 | 10.84 |  | -1.74 | -3.10 | .002 |
| WISC-R Mazes |  | 1262 | 14.03 | 4.99 | 0.00 | 45.00 |  | 14.12 | 4.90 |  | 1.74 | 6.85 | < .001 |
| **Cognitive functioning (age 17 years)** |  |  |  |  |  |  |  |  |  |  |  |  |  |
| TRAIL A mean time |  | 823 | 2.28 | 0.52 | 1.16 | 6.37 |  | 2.30 | 0.52 |  | -0.09 | -2.99 | .003 |
| TRAIL B mean time |  | 823 | 3.06 | 0.91 | 1.36 | 8.35 |  | 3.12 | 0.92 |  | -0.11 | -2.04 | .042 |
| PMT largest maze correct |  | 823 | 11.07 | 2.41 | 3.00 | 19.00 |  | 10.94 | 2.30 |  | 1.12 | 8.75 | < .001 |
| PMT processing speed |  | 823 | 1.92 | 1.00 | 0.20 | 8.37 |  | 1.86 | 0.95 |  | 0.47 | 8.72 | < .001 |
| PMT inspection speed |  | 823 | 8.79 | 11.18 | 1.00 | 100.00 |  | 8.54 | 10.75 |  | 1.83 | 3.00 | .003 |

*Note.* Positive values for mean difference indicate higher means in males whereas negative values indicate higher means in females.

Table S4. Zero-Order Correlations for Cognitive Functioning Indicators at Ages 3, 11, and 17 Years Based on the Imputed Data.

|  | Variable | 1 | 2 | 3 | 4 | 5 | 6 | 7 | 8 | 9 | 10 | 11 | 12 | 13 | 14 | 15 | 16 | 17 | 18 | 19 |
| --- | --- | --- | --- | --- | --- | --- | --- | --- | --- | --- | --- | --- | --- | --- | --- | --- | --- | --- | --- | --- |
| 1 | BTBC-P Building Objects |  |  |  |  |  |  |  |  |  |  |  |  |  |  |  |  |  |  |  |
| 2 | BTBC-P Copy Designs | .49** |  |  |  |  |  |  |  |  |  |  |  |  |  |  |  |  |  |  |
| 3 | BTBC-P Size/Length/Number Discriminations | .55** | .44** |  |  |  |  |  |  |  |  |  |  |  |  |  |  |  |  |  |
| 4 | BTBC-P Info/Labels | .63** | .50** | .63** |  |  |  |  |  |  |  |  |  |  |  |  |  |  |  |  |
| 5 | BTBC-P Name Colors | .32** | .36** | .48** | .48** |  |  |  |  |  |  |  |  |  |  |  |  |  |  |  |
| 6 | BTBC-P Similarities/Differences | .46** | .43** | .54** | .53** | .39** |  |  |  |  |  |  |  |  |  |  |  |  |  |  |
| 7 | TRAIL 11 A mean time | -.10** | -.09** | -.09** | -.11** | -.09** | -.08** |  |  |  |  |  |  |  |  |  |  |  |  |  |
| 8 | TRAIL 11 B mean time | -.02 | -.04 | -.06* | .00 | -.04 | .00 | .07* |  |  |  |  |  |  |  |  |  |  |  |  |
| 9 | WISC-R Similarities | .20** | .20** | .27** | .26** | .23** | .20** | -.22** | -.06* |  |  |  |  |  |  |  |  |  |  |  |
| 10 | WISC-R Digit Span | .13** | .15** | .18** | .17** | .19** | .14** | -.32** | .02 | .40** |  |  |  |  |  |  |  |  |  |  |
| 11 | WISC-R Picture Completion | .16** | .15** | .17** | .19** | .17** | .15** | -.26** | -.06* | .37** | .29** |  |  |  |  |  |  |  |  |  |
| 12 | WISC-R Block Design | .14** | .20** | .18** | .17** | .23** | .13** | -.32** | -.18** | .43** | .37** | .49** |  |  |  |  |  |  |  |  |
| 13 | WISC-R Object Assembly | .16** | .21** | .18** | .21** | .22** | .16** | -.32** | -.13** | .38** | .34** | .48** | .63** |  |  |  |  |  |  |  |
| 14 | WISC-R Coding | .17** | .21** | .20** | .18** | .19** | .15** | -.41** | -.08** | .37** | .47** | .32** | .44** | .38** |  |  |  |  |  |  |
| 15 | WISC-R Mazes | .17** | .16** | .15 | .15** | .13** | .10** | -.40** | -.08** | .31** | .39** | .36** | .48** | .49** | .44** |  |  |  |  |  |
| 16 | TRAIL 17 A mean time | -.07* | -.06* | -.09** | -.06+ | -.11** | -.06+ | .32** | .08* | -.27** | -.33** | -.24** | -.33** | -.33** | -.34** | -.37** |  |  |  |  |
| 17 | TRAIL 17 B mean time | -.15** | -.14** | -.16** | -.13** | -.16** | -.09** | .39** | .08* | -.35** | -.42** | -.28** | -.39** | -.35** | -.44** | -.45** | .74** |  |  |  |
| 18 | PMT largest maze correct | .10** | .15** | .13** | .12** | .13** | .09** | -.27** | -.09* | .32** | .33** | .34** | .51** | .48** | .34** | .44** | -.46** | -.44** |  |  |
| 19 | PMT processing speed | .09** | .11** | .12** | .10** | .12** | .08** | -.23** | -.11** | .28** | .31** | .34** | .48** | .43** | .28** | .39** | -.42** | -.40** | .82** |  |
| 20 | PMT inspection speed | .03 | .00 | .06* | .02 | .04 | .03 | -.03 | -.11** | .14** | .08* | .11** | .20** | .18** | .11** | .06+ | -.19** | -.16** | .41** | .50** |

*Note*. *N*= 1795. BTBC-P = Boehm Test of Basic Concepts – Preschool Version; PMT = Perceptual Maze Test; TRAIL 11 = Trail Making Test assessed at age 11 years; TRAIL 17 = Trail Making Test assessed at age 17 years; WISC-R = Wechsler Intelligence Scale for Children – Revised.

+ *p* < .10. * *p* < .05. ** *p* < .01.

**Table S5. Standardized and Unstandardized Coefficients for Models of Cognitive Functioning at Ages 3,11 and 17 Years Based on Imputed Data.**

|  | ***Unstand.  estimates*** | | ***Stand. estimates*** |  |  | ***Unstand.  estimates*** | | ***Stand. estimates*** |  |  | ***Unstand.  estimates*** | | ***Stand. estimates*** |
| --- | --- | --- | --- | --- | --- | --- | --- | --- | --- | --- | --- | --- | --- |
|  | ***b*** | ***SE*** |  |  |  | ***b*** | ***SE*** |  |  |  | ***b*** | ***SE*** |  |
| **Age 3 years** |  |  |  |  |  |  |  |  |  |  |  |  |  |
| Cognitive composite |  |  |  |  | Performance-IQ |  |  |  |  |  |  |  |  |
| BTBC-P Information | 1.00^+^ |  | .827 |  | BTBC-P Geometric Design | 1.00^+^ |  | .646 |  |  |  |  |  |
| BTBC-P Similarities | 0.15 | 0.01 | .663 |  | BTBC-P Block Design | 1.25 | 0.05 | .765 |  |  |  |  |  |
| BTBC-P Arithmetic | 0.26 | 0.01 | .769 |  | Verbal-IQ |  |  |  |  |  |  |  |  |
| BTBC-P Colors | 0.22 | 0.01 | .568 |  | BTBC-P Information | 1.00^+^ |  | .829 |  |  |  |  |  |
| BTBC-P Geometric Design | 0.12 | 0.01 | .620 |  | BTBC-P Similarities | 0.15 | 0.01 | .665 |  |  |  |  |  |
| BTBC-P Block Design | 0.15 | 0.01 | .726 |  | BTBC-P Arithmetic | 0.26 | 0.01 | .773 |  |  |  |  |  |
|  |  |  |  |  | BTBC-P Colors | 0.22 | 0.01 | .572 |  |  |  |  |  |
| **Age 11 years** |  |  |  |  |  |  |  |  |  |  |  |  |  |
| Cognitive composite |  |  |  |  | TRAIL |  |  |  |  | TRAIL |  |  |  |
| WISC-R Object Assembly | 1.00^+^ |  | .734 |  | TRAIL A mean time | 1.00^+^ |  | .498 |  | TRAIL A mean time | 1.00^+^ |  | .473 |
| WISC-R Block Design | 1.62 | 0.07 | .766 |  | TRAIL B mean time | 1.13 | 0.27 | .149 |  | TRAIL B mean time | 1.20 | 0.27 | .154 |
| WISC-R Coding | 1.39 | 0.08 | .620 |  | IQ |  |  |  |  | Verbal-IQ |  |  |  |
| WISC-R Mazes | 0.6 | 0.04 | .654 |  | WISC-R Object Assembly | 1.00^+^ |  | .734 |  | WISC-R Similarities | 1.00^+^ |  | .632 |
| WISC-R Picture Completion | 0.33 | 0.02 | .599 |  | WISC-R Block Design | 1.62 | 0.07 | .766 |  | WISC-R Digit Span | 0.50 | 0.04 | .626 |
| WISC-R Similarities | 0.44 | 0.03 | .556 |  | WISC-R Coding | 1.39 | 0.08 | .620 |  | Performance-IQ |  |  |  |
| WISC-R Digit Span | 0.22 | 0.01 | .555 |  | WISC-R Mazes | 0.66 | 0.03 | .654 |  | WISC-R Object Assembly | 1.00^+^ |  | .739 |
| TRAIL A mean time | -0.21 | 0.01 | -.487 |  | WISC-R Picture Completion | 0.33 | 0.02 | .599 |  | WISC-R Block Design | 1.61 | 0.07 | .770 |
| TRAIL B mean time | -0.23 | 0.05 | -.149 |  | WISC-R Similarities | 0.44 | 0.03 | .556 |  | WISC-R Coding | 1.37 | 0.08 | .616 |
|  |  |  |  |  | WISC-R Digit Span | 0.22 | 0.01 | .555 |  | WISC-R Mazes | 0.65 | 0.03 | .656 |
|  |  |  |  |  |  |  |  |  |  | WISC-R Picture Completion | 0.33 | 0.02 | .600 |
|  |  |  |  |  |  |  |  |  |  |  |  |  |  |
| **Age 17 years** |  |  |  |  |  |  |  |  |  |  |  |  |  |
| Cognitive composite |  |  |  |  | TRAIL |  |  |  |  |  |  |  |  |
| PMT max. number of rows | 1.00^+^ |  | .903 |  | TRAIL A mean time | 1.00^+^ |  | .881 |  |  |  |  |  |
| PMT processing speed | 0.12 | 0.02 | .900 |  | TRAIL B mean time | 1.71 | 0.13 | .840 |  |  |  |  |  |
| PMT inspection speed | 2.57 | 0.23 | .491 |  | PMT |  |  |  |  |  |  |  |  |
| TRAIL A mean time | -0.13 | 0.01 | -.517 |  | PMT max. number of rows | 1.00^+^ |  | .883 |  |  |  |  |  |
| TRAIL B mean time | -0.22 | 0.02 | -.499 |  | PMT processing speed | 0.43 | 0.02 | .929 |  |  |  |  |  |
|  |  |  |  |  | PMT inspection speed | 2.71 | 0.24 | .505 |  |  |  |  |  |

*Note*. *N* = 1795. ^+^ fixed parameters. Unstand. estimates = unstandardized estimates. Stand. estimates = standardized estimates. BTBC-P = Boehm Test of Basic Concepts – Preschool Version (Boehm, 1986), TRAIL = Trail Making Test (Levander & Elithorn, 1987), WISC-R = Wechsler Intelligence Scale for Children - Revised (Wechsler, 1974), PMT = Perceptual Maze Test (Levander & Elithorn, 1987).

All *p*s < .001.

**Table S6. Regression Results for Cognitive Functioning at Ages 3, 11 and 17 Years Predicted by Early Childhood Risk Factors (Age 3 Years), Sex, and Previous Cognitive Performance.**

|  | **Cognitive Functioning**  **(Age 3 Years)** | | | |  | **Cognitive Functioning**  **(Age 11 Years)** | | | |  | **Trial Making Test Performance**  **(Age 17 Years)** | | | |  | **Perceptual Maze Test Performance**  **(Age 17 Years)** | | | |
| --- | --- | --- | --- | --- | --- | --- | --- | --- | --- | --- | --- | --- | --- | --- | --- | --- | --- | --- | --- |
| Predictor | *b* | *SE* | *β* | *p* |  | *b* | *SE* | *β* | *p* |  | *b* | *SE* | *β* | *p* |  | *b* | *SE* | *β* | *p* |
| (Intercept) | -0.26 | 0.11 |  | .021 |  | -0.34 | 0.09 |  | <.001 |  | 0.11 | 0.16 | 0.00 | .511 |  | 0.34 | 0.15 |  | .022 |
| Years school father | 0.01 | 0.01 | 0.10 | .414 |  | 0.01 | 0.01 | 0.07 | .037 |  | 0.01 | 0.01 | 0.02 | .529 |  | -0.01 | 0.01 | -0.04 | .259 |
| Years school mother | 0.01 | 0.01 | 0.03 | .185 |  | 0.02 | 0.01 | 0.14 | .004 |  | -0.02 | 0.01 | -0.06 | .111 |  | -0.02 | 0.01 | -0.05 | .151 |
| Occupational status father | 0.04 | 0.02 | 0.06 | .021 |  | 0.03 | 0.02 | 0.09 | .084 |  | -0.02 | 0.03 | -0.05 | .364 |  | 0.03 | 0.02 | 0.01 | .289 |
| Occupational status mother | 0.03 | 0.02 | 0.09 | .086 |  | 0.04 | 0.01 | 0.07 | .007 |  | -0.01 | 0.02 | -0.01 | .735 |  | -0.01 | 0.02 | -0.01 | .528 |
| Number of people per room | -0.03 | 0.01 | -0.04 | .018 |  | -0.02 | 0.01 | -0.07 | .063 |  | 0.02 | 0.02 | 0.03 | .327 |  | -0.02 | 0.02 | -0.02 | .343 |
| Condition of house | 0.00 | 0.05 | 0.02 | .918 |  | 0.11 | 0.04 | 0.08 | .003 |  | 0.02 | 0.07 | 0.02 | .743 |  | -0.02 | 0.06 | 0.01 | .749 |
| Anaemia | -0.05 | 0.02 | -0.11 | .010 |  | -0.02 | 0.02 | -0.05 | .145 |  | 0.03 | 0.03 | 0.03 | .223 |  | 0.02 | 0.03 | -0.01 | .433 |
| Stunting | -0.07 | 0.03 | -0.09 | .010 |  | -0.11 | 0.02 | -0.16 | <.001 |  | -0.02 | 0.04 | -0.01 | .699 |  | 0.01 | 0.04 | 0.00 | .784 |
| BTBC-P | - | - | - | - |  | 0.18 | 0.03 | 0.21 | <.001 |  | -0.01 | 0.05 | -0.01 | .807 |  | 0.01 | 0.05 | 0.00 | .750 |
| TRAIL 11 | - | - | - | - |  | - | - | - | - |  | 0.15 | 0.06 | 0.14 | .019 |  | 0.02 | 0.06 | 0.04 | .729 |
| WISC-R | - | - | - | - |  | - | - | - | - |  | 0.70 | 0.06 | 0.51 | <.000 |  | 0.67 | 0.06 | 0.46 | <.001 |
| Sex:female | -0.14 | 0.16 | 0.05 | .381 |  | -0.14 | 0.13 | -0.14 | .270 |  | -0.01 | 0.22 | -0.03 | .956 |  | -0.50 | 0.20 | -0.28 | .011 |
| Years school father * sex | 0.03 | 0.01 | 0.07 | .015 |  | 0.00 | 0.01 | -0.02 | .624 |  | 0.00 | 0.02 | 0.00 | .852 |  | 0.01 | 0.01 | 0.01 | .674 |
| Years school mother * sex | -0.01 | 0.01 | -0.02 | .558 |  | 0.01 | 0.01 | 0.03 | .275 |  | 0.00 | 0.02 | 0.01 | .804 |  | 0.01 | 0.02 | 0.01 | .635 |
| Occupational status father * sex | -0.03 | 0.02 | -0.03 | .210 |  | 0.01 | 0.02 | 0.02 | .475 |  | -0.02 | 0.03 | -0.02 | .626 |  | -0.04 | 0.03 | -0.04 | .172 |
| Occupational status mother * sex | 0.04 | 0.03 | 0.03 | .120 |  | -0.02 | 0.02 | -0.02 | .311 |  | 0.01 | 0.03 | 0.00 | .854 |  | 0.01 | 0.03 | 0.01 | .631 |
| Number of people per room * sex | 0.03 | 0.02 | 0.03 | .108 |  | -0.01 | 0.02 | -0.01 | .656 |  | -0.01 | 0.03 | 0.00 | .847 |  | 0.02 | 0.02 | 0.02 | .458 |
| Condition of house * sex | 0.04 | 0.07 | 0.02 | .515 |  | -0.03 | 0.05 | -0.01 | .565 |  | 0.03 | 0.09 | 0.01 | .709 |  | 0.05 | 0.08 | 0.02 | .493 |
| Anaemia * sex | -0.03 | 0.03 | -0.02 | .339 |  | -0.01 | 0.02 | -0.01 | .782 |  | -0.01 | 0.03 | 0.00 | .664 |  | -0.05 | 0.03 | -0.03 | .138 |
| Stunting * sex | -0.01 | 0.04 | -0.01 | .806 |  | 0.00 | 0.03 | 0.00 | .997 |  | 0.01 | 0.05 | 0.00 | .919 |  | -0.02 | 0.04 | -0.01 | .715 |
| BTBC-P * sex | - | - | - | - |  | 0.00 | 0.04 | 0.00 | .946 |  | 0.02 | 0.07 | 0.01 | .779 |  | -0.02 | 0.06 | -0.01 | .705 |
| TRAIL 11 * sex | - | - | - | - |  | - | - | - | - |  | 0.05 | 0.08 | 0.02 | .520 |  | 0.04 | 0.06 | 0.02 | .523 |
| WISC-R * sex | - | - | - | - |  | - | - | - | - |  | -0.03 | 0.09 | -0.01 | .731 |  | -0.24 | 0.08 | -0.10 | .002 |
|  |  |  |  |  |  |  |  |  |  |  |  |  |  |  |  |  |  |  |  |
|  | *R^2^*  = .098 \| R²_adj_ = .090 | | | |  | R2 = .290 \| R²adj = .283 (p < .001) | | | |  | *R^2^* = .319 \| *R²_adj_* = .310 (*p* < .001) | | | |  | *R^2^* = .344 \| *R²_adj_* = .335 (*p* < .001) | | | |
|  | 2.043 (*p* = .032) | | | |  | 0.361 (*ns*) | | | |  | 0.127 (ns) | | | |  | 1.676 (*ns*) | | | |

*Note. ns* = not significant.

**Table S7. Correlations Between Cognitive Functioning Composite Variables at Ages 3, 11 and 17 Years and Early Childhood Risk Factors Assessed at Age 3 Years for the Imputed Data.**

|  | **Age 3 years** |  | **Age 11 years** | | |  | **Age 17 years** | | |
| --- | --- | --- | --- | --- | --- | --- | --- | --- | --- |
| **Early childhood risk factors** | **COG** |  | **COG** | WISC-R | TMT |  | **COG** | PMT | TMT |
|  |  |  |  |  |  |  |  |  |  |
| Years school father | **.19**** |  | **.30**** | .31** | .11** |  | **.14**** | .10** | .14** |
| Years school mother | **.18**** |  | **.33**** | .34** | .15** |  | **.12**** | .10** | .12** |
| Occupational status father | **.16**** |  | **.28**** | .28** | .15** |  | **.13**** | .12** | .10** |
| Occupational status mother | **.13**** |  | **.16**** | .16** | .05+ |  | **.06*** | .04 | .06* |
| Number of people per room | **-.10**** |  | **-.19**** | -.18** | -.11** |  | **-.10**** | -.09** | -.07* |
| Condition of house | **.09**** |  | **.21**** | .21** | .12** |  | **.13**** | .10** | .12** |
| Anaemia | **-.18**** |  | **-.21**** | -.21** | -.10** |  | **-.11**** | -.10** | -.07* |
| Stunting | **-.17**** |  | **-.32**** | -.33** | -.13** |  | **-.21**** | -.17** | -.18** |

*Note*. *N* = 1795*.* Positive values for mean composites indicate higher cognitive performance. COG = cognitive functioning composite, TMT = Trail Making Test (Levander & Elithorn, 1987), WISC-R = Wechsler Intelligence Scale for Children - Revised (Wechsler, 1974), PMT = Perceptual Maze Test (Levander & Elithorn, 1987).

+ *p* < .10. * *p* < .05. ** *p* < .01.
