# Supplementary material for: Associations between early childhood poverty and cognitive functioning throughout childhood and adolescence: A 14-year prospective longitudinal analysis of the Mauritius Child Health Project
Source: PLoS One. 2023 Feb 24;18(2):e0278618. doi: 10.1371/journal.pone.0278618 (PMC9956590; doi:10.1371/journal.pone.0278618)
Supplement: S2 File — (DOCX) [file pone.0278618.s002.docx]

**Supplemental Material on Latent Growth Curve Modelling**

Latent growth curve modeling provides a flexible framework to examine both intraindividual change over time and interindividual differences in these trajectories, accounting for complexities such as missing or non-normally distributed data or unequally spaced assessments [1,2]. For the subsequent analyses, cognitive functioning composites at ages 3, 11 and 17 years were based on the one-factor models, with estimates being computed as the means of the respective standardized indicators (cf. *Confirmatory factor analysis* in the main manuscript).

In a first step, a linear latent growth curve model (LGCM) with constrained residuals was compared to a LGCM with residual variance freely estimated across time. In a second step, sex and risk factors were added as predictors of both intercept and slope. The intercept indicates the initial mean level in cognitive functioning (i.e., at age 3 years), and the slope represents the average degree of linear change in cognitive performance over time (i.e., across ages 3, 11 and 17 years). Loadings on the intercept were fixed to 1 for each wave to represent a constant across time, and loadings on the slope were defined on the basis of time in years between assessments, that is, 0, 8, and 14, the intercept being defined at age 3 years. Intercept and slope were allowed to covary. Model parameters were estimated by using full information maximum likelihood. Models were specified using the *growth()* function of the R package *lavaan* (version 0.6-12; [3])

For model fit, we report the comparative fit index (CFI) and the Tucker-Lewis index (TLI). Values of .90 and above are generally taken to indicate acceptable fit and values of .95 and above to indicate good fit. In addition, root mean square error of approximation (RMSEA) and standardized root mean square residual (SRMR) are reported. We adopted the criteria of .08 and below as indicating good fit for SRMR and values of .06 and below as indicating acceptable fit for RMSEA (e.g., [5–7]). To compare the fit of non-nested models, we analyzed differences in Bayesian information criterion (BIC) values and χ^2^ [8].

## Results

In examining the effects over time, we found that a two-parameter growth model with unconstrained residuals (*N* = 1779, χ^2^(1) = 24.23, *p* < .001, *BIC* = 7730.197) fit the data significantly better than the one with constrained residuals, *N* = 1,779; χ^2^(3) = 62.92, *p*< .001, *BIC* = 7706.47), with Δχ^2^ = 38.69, *p* < .001. In both models, intercept as well as slope accounted for variance significantly, suggesting individual differences in initial values and in linear change. The better fit of the unconstrained residuals model indicates that error variances vary across timepoints, which was somewhat anticipated given the fact that different measures were used. Hence, the unconstrained residual model was used as a basis for the subsequent analyses.

To examine the associations between early childhood risk factors and cognitive trajectories, we ran two conditional growth curve models. First, we included risk factors assessed at age 3 years and sex as predictors for both initial and change levels. Second, sex was removed as a predictor, and a multigroup model with the risk factors as predictors for intercept and slope was fitted separately for males and females so that we could investigate whether associations differed across the sexes. Both models showed an acceptable fit, with CFI > .90, RMSEA < .07, and SRMR < .03 (see Tables S01 and S02, for fit indices and model parameters). Only TLI estimates were below the acceptable range, which might be caused by the unconstrained error variances as with TLI the model fit worsens as the number of model indicators increases [4].

Overall, results indicated that risk factors were significantly associated with cognitive functioning at age 3 years but did not significantly predict cognitive trajectories over time (see Table S01). A converse pattern, however, could be observed for sex, with girls starting off with slightly higher intercepts at age 3 years (*β*= 0.14, *p* < .001) but then declining significantly over time (*β*= -0.44, *p* < .001), regardless of poverty-related risks experienced at early age. There was no significant covariance between intercept and slope factors, indicating that change in mean levels was independent from initial mean levels. The best predictors of initial cognitive functioning were malnutrition indicators as well as parental occupational and educational attainment (Table S01).

**Table S01. Associations Between Poverty-Related Risk Factors and Sex and the Initial and Change Levels of Cognitive Functioning Based on the Linear Latent Growth Model.**

|  |  | Linear LGCM | | | | |
| --- | --- | --- | --- | --- | --- | --- |
| χ² (*df*) |  | 70.647*** (10) | | | | |
| CFI |  | .926 | | | | |
| TLI |  | .778 | | | | |
| AIC |  | 6030.232 | | | | |
| BIC |  | 6168.514 | | | | |
| RMSEA [90% CI] |  | .063 *ns*[.050, .078] | | | | |
| SRMR |  | .026 | | | | |
|  |  |  |  |  |  |  |
|  |  | *β* | *B* | *SE* | *z* | *p* |
| **Intercept** |  |  |  |  |  |  |
| Occupational status mother |  | 0.17 | 0.05 | 0.01 | 3.85 | <.001 |
| Occupational status father |  | 0.13 | 0.03 | 0.01 | 2.51 | .012 |
| Years school mother |  | 0.13 | 0.02 | 0.01 | 2.58 | .010 |
| Years school father |  | 0.20 | 0.02 | 0.01 | 3.70 | <.001 |
| Number of people per room |  | -0.10 | -0.02 | 0.01 | -2.12 | .034 |
| Condition of house |  | 0.03 | 0.02 | 0.04 | 0.70 | .482 |
| Stunting |  | -0.23 | -0.09 | 0.02 | -4.86 | <.001 |
| Anemia |  | -0.23 | -0.07 | 0.01 | -5.05 | <.001 |
| Sex:female |  | 0.14 | 0.11 | 0.04 | 3.08 | <.001 |
|  |  |  |  |  |  |  |
| **Slope** |  |  |  |  |  |  |
| Occupational status mother |  | -0.06 | 0.00 | 0.00 | -1.07 | .283 |
| Occupational status father |  | 0.02 | 0.00 | 0.00 | 0.36 | .719 |
| Years school mother |  | 0.01 | 0.00 | 0.00 | 0.22 | .825 |
| Years school father |  | -0.07 | 0.00 | 0.00 | -1.13 | .259 |
| Number of people per room |  | -0.02 | 0.00 | 0.00 | -0.37 | .713 |
| Condition of house |  | 0.08 | 0.01 | 0.00 | 1.48 | .140 |
| Stunting |  | -0.07 | 0.00 | 0.00 | -1.22 | .223 |
| Anemia |  | 0.09 | 0.00 | 0.00 | 1.70 | .089 |
| Sex:female |  | -0.44 | -0.04 | 0.00 | -8.59 | <.001 |

*Note*.  CFI = comparative fit index; TLI = Tucker-Lewis index; AIC = Akaike information criterion; BIC = Bayesian information criterion; RMSEA = root mean square error of approximation; 90% CI = 90% confidence interval for population RMSE; SRMR = standardized root mean square residual.

****p* < .001. *ns* = nonsignificant.

A similar pattern emerged when analyses were computed separately for males and females, with risk factors predicting initial cognitive functioning but not developmental trajectories (see Table S02). Intercept and slope variance was approximately the same for boys and girls, indicating that individual trajectories differed to a similar extent within both groups. No significant interactions between intercept and slope were found for either boys or girls, indicating that initial cognitive functioning at age 3 years is not related to further cognitive development throughout childhood and adolescence. Again, malnutrition indicators and parental education were found to be the best predictors of initial mean levels of cognitive functioning, with similar-sized effects for boys and girls.

**Table S02. Associations Between Poverty-Related Risk Factors and the Initial and Change Levels of Cognitive Functioning Based on the Multigroup Latent Growth Model by Sex.**

|  |  | Multigroup model | | | | | | | | | | |
| --- | --- | --- | --- | --- | --- | --- | --- | --- | --- | --- | --- | --- |
| χ² (*df*) |  | 80.588*** (18) | | | | | | | | | | |
| CFI |  | .914 | | | | | | | | | | |
| TLI |  | .741 | | | | | | | | | | |
| AIC |  | 6028.964 | | | | | | | | | | |
| BIC |  | 6284.254 | | | | | | | | | | |
| RMSEA [90% CI] |  | .068* [.053, .083] | | | | | | | | | | |
| SRMR |  | .030 | | | | | | | | | | |
| *N* |  | Males (*N*= 777) | | | | |  | Females (*N*= 731) | | | | |
|  |  | β | *B* | *SE* | *z* | *p* |  | β | *B* | *SE* | *z* | *p* |
| Intercept |  |  |  |  |  |  |  |  |  |  |  |  |
| Occupational status, mother |  | 0.13 | 0.04 | 0.02 | 1.88 | .060 |  | 0.20 | 0.07 | 0.02 | 3.41 | <.001 |
| Occupational status, father |  | 0.15 | 0.03 | 0.02 | 1.87 | .061 |  | 0.11 | 0.03 | 0.02 | 1.62 | .105 |
| Years school, mother |  | 0.19 | 0.02 | 0.01 | 2.36 | .018 |  | 0.08 | 0.01 | 0.01 | 1.23 | .219 |
| sYears school, father |  | 0.09 | 0.01 | 0.01 | 1.06 | .291 |  | 0.29 | 0.04 | 0.01 | 4.23 | <.001 |
| Number of people per room |  | -0.16 | -0.03 | 0.01 | -2.30 | .021 |  | -0.05 | -0.01 | 0.02 | -0.88 | .381 |
| Condition of house |  | 0.03 | 0.02 | 0.05 | 0.47 | .637 |  | 0.04 | 0.03 | 0.05 | 0.66 | .507 |
| Stunting |  | -0.24 | -0.09 | 0.03 | -3.39 | .001 |  | -0.21 | -0.09 | 0.03 | -3.46 | .001 |
| Anemia |  | -0.22 | -0.06 | 0.02 | -3.08 | .002 |  | -0.25 | -0.08 | 0.02 | -4.11 | <.001 |
|  |  |  |  |  |  |  |  |  |  |  |  |  |
| Slope |  |  |  |  |  |  |  |  |  |  |  |  |
| Occupational status, mother |  | 0.02 | 0.00 | 0.00 | 0.21 | .834 |  | -0.10 | 0.00 | 0.00 | -1.37 | .170 |
| Occupational status, father |  | 0.09 | 0.00 | 0.00 | 0.91 | .363 |  | -0.07 | 0.00 | 0.00 | -0.81 | .419 |
| Years school, mother |  | -0.09 | 0.00 | 0.00 | -0.82 | .413 |  | 0.07 | 0.00 | 0.00 | 0.86 | .390 |
| Years school, father |  | 0.08 | 0.00 | 0.00 | 0.71 | .480 |  | -0.20 | 0.00 | 0.00 | -2.24 | .025 |
| Number of people per room |  | 0.01 | 0.00 | 0.00 | 0.13 | .900 |  | -0.03 | 0.00 | 0.00 | -0.38 | .701 |
| Condition of house |  | 0.08 | 0.01 | 0.01 | 0.88 | .377 |  | 0.10 | 0.01 | 0.01 | 1.33 | .184 |
| Stunting |  | -0.14 | -0.01 | 0.00 | -1.49 | .137 |  | -0.01 | 0.00 | 0.00 | -0.14 | .888 |
| Anemia |  | 0.15 | 0.00 | 0.00 | 1.70 | .089 |  | 0.05 | 0.00 | 0.00 | 0.68 | .500 |

*Note*.  CFI = comparative fit index; TLI = Tucker-Lewis index; AIC = Akaike information criterion; BIC = Bayesian information criterion; RMSEA = root mean square error of approximation; 90% CI = 90% confidence interval for population RMSE; SRMR = standardized root mean square residual.

* *p* < .05. ****p* < .001.

References

**1**. McArdle JJ. Latent variable modeling of differences and changes with longitudinal data. Annu Rev Psychol. 2009; 60:577–605. doi: 10.1146/annurev.psych.60.110707.163612 PMID: 18817479.

**2**. Curran PJ, Obeidat K, Losardo D. Twelve Frequently Asked Questions About Growth Curve Modeling. J Cogn Dev. 2010; 11:121–36. doi: 10.1080/15248371003699969 PMID: 21743795.

**3**. Rosseel Y. lavaan: An R Package for Structural Equation Modeling. J Stat Soft. 2012; 48. doi: 10.18637/jss.v048.i02.

**4**. Shi D, Lee T, Maydeu-Olivares A. Understanding the Model Size Effect on SEM Fit Indices. Educ Psychol Meas. 2019; 79:310–34. Epub 2018/06/29. doi: 10.1177/0013164418783530 PMID: 30911195.

**5**. Browne MW, Cudeck R. Alternative Ways of Assessing Model Fit. Sociol Methods Res. 1992; 21:230–58. doi: 10.1177/0049124192021002005.

**6**. Brown TA. Confirmatory factor analysis for applied research. 2nd ed. New York: Guilford Press; 2015.

**7**. Hu L, Bentler PM. Cutoff criteria for fit indexes in covariance structure analysis: Conventional criteria versus new alternatives. Struct Equ Modeling. 1999; 6:1–55. doi: 10.1080/10705519909540118.

**8**. Raftery AE. Bayesian Model Selection in Social Research. Sociol Methodol. 1995; 25:111–63. doi: 10.2307/271063.
